# An adapted Black Widow Optimization Algorithm for Financial Portfolio Optimization Problem with cardinalty and budget constraints

**DOI:** 10.1038/s41598-024-71193-w

**Published:** 2024-09-28

**Authors:** Rahenda Khodier, Ahmed Radi, Basel Ayman, Mohamed Gheith

**Affiliations:** 1 Department of Industrial and Manufacturing Engineering, Egypt-Japan University of Science and Technology, Alexandria, 21934 Egypt; 2 Production Engineering Department, Alexandria University, Alexandria, 21544 Egypt

**Keywords:** Portfolio optimization, Black Widow Algorithm for Portfolio Optimization, Mean-variance, Meta-heuristic, Engineering, Mathematics and computing

## Abstract

Financial Portfolio Optimization Problem (FPOP) is a cornerstone in quantitative investing and financial engineering, focusing on optimizing assets allocation to balance risk and expected return, a concept evolving since Harry Markowitz’s 1952 Mean-Variance model. This paper introduces a novel meta-heuristic approach based on the Black Widow Algorithm for Portfolio Optimization (BWAPO) to solve the FPOP. The new method addresses three versions of the portfolio optimization problems: the unconstrained version, the equality cardinality-constrained version, and the inequality cardinality-constrained version. New features are introduced for the BWAPO to adapt better to the problem, including (1) mating attraction and (2) differential evolution mutation strategy. The proposed BWAPO is evaluated against other metaheuristic approaches used in portfolio optimization from literature, and its performance demonstrates its effectiveness through comparative studies on benchmark datasets using multiple performance metrics, particularly in the unconstrained Mean-Variance portfolio optimization version. Additionally, when encountering cardinality constraint, the proposed approach yields competitive results, especially noticeable with smaller datasets. This leads to a focused examination of the outcomes arising from equality versus inequality cardinality constraints, intending to determine which constraint type is more effective in producing portfolios with higher returns. The paper also presents a comprehensive mathematical model that integrates real-world constraints such as transaction costs, transaction lots, and a dollar-denominated budget, in addition to cardinality and bounding constraints. The model assesses both equality/inequality cardinality constraint versions of the problem, revealing that the inequality constraint tends to offer a wider range of feasible solutions with increased return potential.

## Introduction

In today’s complex and dynamic world of finance, a substantial volume of capital, amounting to billions of dollars, is invested in diverse economic sectors by a spectrum of market participants, including individual investors, brokerage entities, and fund management professionals^1^. This makes choosing the right assets for investment very important. The goal is to make profits in different market situations and to minimize the losses as much as possible when markets go down. A widely used strategy for achieving this is to create a portfolio with diversified assets, as it helps in spreading out the risk. Portfolio optimization, therefore, plays a crucial role in resources allocation, providing a structured way to maximize returns while minimizing risk^2^.

**Figure 1:**
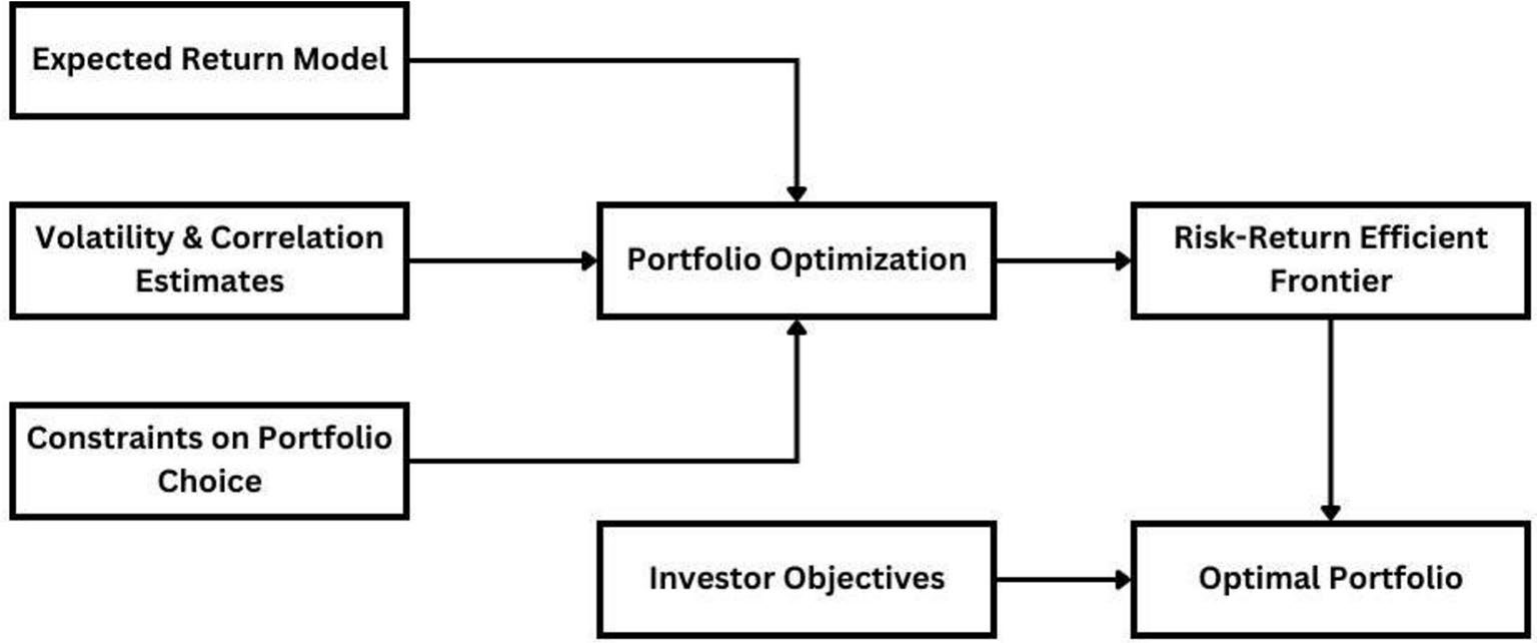
The investment process according to MPT.

In the early 1950s, Harry Markowitz introduced the concept of portfolio optimization by proposing the Mean-Variance (MV) model, which laid the foundation of Modern Portfolio Theory (MPT) based on the concept of the efficient frontier. The MPT represents the tradeoff between return and risk^3^, where investors can select portfolios based on their preferences for risk and return as well as benefiting from diversification as a risk mitigation strategy. The investment process according to MPT is demonstrated in Fig. 1. The inputs required to build an optimized portfolio are the expected return model, the volatility/risk, the correlation between securities/assets, and the real-life constraints. The output is the risk-efficient frontier, which is a tradeoff frontier based on each investor’s objective^4^.

While the MV model remains a cornerstone in quantitative investment strategies, its practical application often encounters limitations due to the absence of explicit real-world constraints in the standard MV framework. Therefore, subsequent research has introduced various realistic constraints to refine the MV model, making it more applicable and relevant to actual investment scenarios such as cardinality, bounding, transaction costs constraints^5,6^, and transaction lots constraint^7–10^. Therefore, three main versions of the FPOP exist: the Unconstrained Portfolio Optimization Problem (UPOP) version, the Equality Cardinality Constrained Portfolio Optimization Problem (CCPOP) version, and the Inequality Cardinality Constraints Portfolio Optimization Problem (ICCPOP) version.

The problem of UPOP is identified as a convex quadratic programming challenge, which can be effectively addressed using linear or quadratic programming. However, when adapting this model to more realistic Portfolio Optimization scenarios, it becomes necessary to introduce additional constraints to the standard framework^11^. These additions, meant to reflect real-world investment considerations, significantly increase the complexity of the problem. Incorporating the cardinality constraint transforms the model from a quadratic optimization model to a Quadratic Mixed Integer model, which is NP-hard. As a result, many heuristic optimization methods have been applied to solve constrained portfolio selection problems under the framework of the mean-variance model^12^.

An extensive review of methodologies used in Mean-Variance Portfolio Optimization (MV-PO) from the 1990s to 2019 highlighted a significant finding: meta-heuristic algorithms were employed in approximately 82% of the studies. This high utilization rate emphasizes meta-heuristics effectiveness in addressing portfolio optimization challenges and showcases their flexibility. These algorithms, capable of handling single and multi-objective scenarios, have been notably prevalent in high-quality research for Cardinality-Constrained Mean-Variance Portfolio Optimization Problems (CCMV-POP). In addition to traditional and meta-heuristic approaches to portfolio optimization, High-Frequency Trading (HFT) has emerged as a significant and influential strategy. HFT involves the use of advanced algorithms and powerful computers to execute a large number of trades at extremely high speeds, often within microseconds or milliseconds. This strategy aims to capitalize on small price discrepancies and market inefficiencies, leveraging high turnover rates and low latency to achieve significant profits. Notable research in HFT focuses on optimal order execution and real-time market data analysis to predict short-term price movements, which propose novel neural networks with softmax equalization technique to address the timely and accurate solution of portfolio selection problems^13^. Additionally, the integration of cardinality constraints in portfolio optimization using neural networks has also been explored in Ref.^14^, highlighting the complexities and advancements in this domain. However, despite the success and popularity of HFT in achieving rapid trading profits, this paper does not adopt HFT methodologies. Instead, a novel meta-heuristic algorithm was employed, the Black Widow Algorithm for Portfolio Optimization (BWAPO), to address the portfolio optimization problem. This approach emphasizes balancing risk and return over a longer time horizon, incorporating real-world constraints such as cardinality, budget, and transaction costs, to provide a robust and diversified investment strategy. The upcoming literature section will provide further details, illustrating the adaptability and efficiency of meta-heuristics in various portfolio optimization contexts^15^.

This paper proposes a novel approach based on the Black Widow Algorithm for Portfolio Optimization (BWAPO) to solve the Portfolio Optimization Problem (POP) that considers cardinality, bounding, and budget constraints. The Black Widow Algorithm for Portfolio Optimization (BWAPO) is a nature-inspired meta-heuristic that has been recently introduced^16^. BWAPO is based on the unique mating and cannibalism behavior of black widow spiders. The results obtained are very promising, demonstrating that the implementation of BWAPO to the POP shows superior performance, reflecting its effectiveness and potential as a cutting-edge solution for these complex financial optimization tasks. This success highlights BWAPO’s capability in navigating and optimizing portfolio management, offering a novel and efficient approach for investors and financial analysts. Furthermore, the paper introduces a mathematical model incorporating cardinality, bounding, budget, transaction cost, and transaction lots constraints.

This paper is organized as follows: section explains the problem, different models adopted for solving POP, and the types of real-world constraints. Section presents a summarized review of the different methods used for POP in the literature and the research gaps. Section demonstrates the proposed BWAPO and the mathematical model for solving the three versions of the POP. Section presents the experimental results. Section provides final observations and conclusions.

## Problem description

The models used in the portfolio optimization problem optimize assets allocation by balancing two critical dimensions: the anticipated return, which is indicated by the mean return of the assets, and the associated risk, measured through the variance of the portfolio for a specified target return. The solution consists of a collection of efficient solutions, collectively forming what is known as the efficient frontier. Each point on this frontier signifies a different equilibrium between risk and return, allowing investors to select a position that best matches their risk and return expectations. The different models addressing POP will be addressed in the following subsections.

### Markowitz mean-variance (MV) model

The original Markowitz Mean-Variance Model, introduced by Harry Markowitz in 1952, is a foundational concept in modern portfolio theory. The model is designed as a single-objective optimization framework, addressing two fundamental aspects of portfolio management: return and risk. It employs variance as a measure of risk to minimize this variance through the objective function Eq. (1) by ensuring that the portfolio has an expected return of *E* as shown in Eq. (2), effectively balancing the trade-off between achieving higher returns and maintaining manageable risk levels. Equations (3) and (4) ensure that the allocation proportions for the chosen assets must be confined within the range [0, 1], ensuring that each asset’s share is positive to prevent short selling and to ensure the investment of all the available capital which equals one^3^.

1 $$\begin{aligned}  &   \text{ min } ~~ ~~&\sum \limits _{i=1}^N \sum \limits _{j=1}^N \sigma _{ij}w_{i}w_{j} \end{aligned}$$

2 $$\begin{aligned}  &   \text{ s.t. } ~~ ~~&\sum \limits _{i=1}^N w_{i}r_{i}=E \end{aligned}$$

3 $$\begin{aligned}  &   \sum \limits _{i=1}^N w_{i}=1 \end{aligned}$$

4 $$\begin{aligned}  &   \sum \limits _{i=1}^N w_{i}\ge 0, ~~ \forall i \in N \end{aligned}$$

where *N* is the total number of assets to choose from, $\sigma _{ij}$ is the covariance between asset *i* and asset *j*, and $w_i$ is the proportion of asset *i* in the portfolio. The average return of asset *i* is represented by $r_i$.

### Cardinality constrained mean-variance (CCMV) model

The CCMV model is derived from the MV model by integrating the risk aversion parameter, which is denoted by $\lambda$. This model involves the incorporation of additional constraints that restrict the number of assets selected and their respective proportions within the portfolio. According to Ref.^17^, the CCMV model is formulated as follows:

5 $$\begin{aligned} \text{ min } ~~ ~~&\lambda \left( \sum \limits _{i=1}^N \sum \limits _{j=1}^N \sigma _{ij}w_{i}w_{j} \right) - (1-\lambda )\sum \limits _{i=1}^N w_{i}r_{i} \end{aligned}$$

6 $$\begin{aligned} \text{ s.t. } ~~ ~~&\sum \limits _{i=1}^N w_{i}=1 \end{aligned}$$

7 $$\begin{aligned}{}&\sum \limits _{i=1}^N x_{i}=K \end{aligned}$$

8 $$\begin{aligned}{}&\varepsilon _i x_i \le w_i \le \delta _i x_i, ~ i=1, ~\dots , ~N \end{aligned}$$

9 $$\begin{aligned}{}&x_i \in \{0, 1\}, ~ i=1, ~\dots , ~N \end{aligned}$$

10 $$\begin{aligned}{}&\lambda \in [0, 1] \end{aligned}$$

where *K* is the exact number of assets to be included in the portfolio, and $\varepsilon _i$, $\delta _i$ is the minimum and maximum proportion of asset *i* to be included in the portfolio, respectively. In the context of Eq. (5), when $\lambda$ takes the value 0, the objective then is to maximize the expected return without regard for the associated risk, leading to an optimal solution that exclusively includes the single asset offering the highest return. Conversely, when $\lambda$ equals one, the objective shifts to minimizing risk irrespective of the returns, typically resulting in a solution that creates a diversified portfolio of multiple assets. For values of $\lambda$ that fall within the range of [0,1], there is a deliberate trade-off between risk and return. This creates solutions between the two extreme scenarios of $\lambda = 0$ and $\lambda = 1$.

### Constraints

The traditional mean-variance mentioned above lacks the real-world constraints that the investors seek in the portfolio. Consequently, various constrained versions of this model have been developed. These additional constraints add considerable complexity to the problem of portfolio management. Below, some of the most frequently encountered constraints in portfolio optimization are discussed.

*Cardinality constraint (CC)* limits the total number of assets in the portfolio to a certain limit^17^. The constraint can also be represented as an Inequality Cardinality constraint (IC)^18,19^.

*Boundary constraint (BC)* which limits the upper and lower bounds on the assets’ weights, ensures that an asset remains balanced within the portfolio, avoiding excessive risk through overrepresentation or diminished impact due to underrepresentation. When both the cardinality and boundary constraints are encountered in the traditional MV model, it is referred to as the Cardinality Constrained Mean-Variance Model (CCMV), presented by^17^.

*Transaction costs (TC)* represent fees that investors incur when buying or selling stocks, directly impacting the total profit. The transaction cost constraint is designed to ensure that these expenses stay within a predefined budget, thus maintaining the overall financial efficacy of the investment^20,21^.

*Rounding to Lot (RL)* specifies the minimum quantity of an asset that can be traded in one transaction. It ensures that the number of units of any invested asset is a multiple of its minimum lot size^22,23^. Table 1 presents an overview of the different studies conducted, encompassing various constraints encountered.

**Table 1:** Comparative overview of previous work and our approach with types of constraints encountered.

Authors	Proposed approach	Constraints				
		CC	IC	BC	TC	RL
Woodside-Oriakhi et al.^24^	Genetic algorithm, Tabu search and Simulated annealing	$\checkmark$		$\checkmark$		
Yin et al.^25^	Improved particle swarm algorithms based on random population topology strategy	$\checkmark$		$\checkmark$		
Kalayci et al.^26^	Artificial bee colony algorithm with feasibility enforcement and infeasibility toleration procedures	$\checkmark$		$\checkmark$		
Bacanin et al.^27^	Modified Firefly Algorithm	$\checkmark$		$\checkmark$		
Dhaini and Mansour^28^	Squirrel Search Algorithm	$\checkmark$		$\checkmark$		
Cura,^29^	Artificial Bee Colony	$\checkmark$		$\checkmark$		
Zheng et al.^15^	Mayfly Algorithm	$\checkmark$		$\checkmark$		
Lwin et al.^30^	Efficient learning-guided hybrid multi-objective evolutionary algorithm	$\checkmark$		$\checkmark$		$\checkmark$
Liagkouras and Metaxiotis^31^	Specially engineered multi-objective evolutionary algorithm (MOEA)	$\checkmark$		$\checkmark$		$\checkmark$
Chiam et al.^32^	Evolutionary multi-objective algorithm	$\checkmark$		$\checkmark$		$\checkmark$
Ruiz-Torrubiano and Suarez^33^	Genetic algorithm (GA) and quadratic programming	$\checkmark$		$\checkmark$	$\checkmark$	
Xu et al.^34^	Improved Particle Swarm Optimization (IPSO)	$\checkmark$		$\checkmark$	$\checkmark$	
This work (CCMV-POP)	Black Widow Algorithm for Portfolio Optimization (BWAPO)	$\checkmark$		$\checkmark$		
This work (Inequality Cardinailty Constrained Mean-variance Model)	Black Widow Algorithm for Portfolio Optimization (BWAPO)		$\checkmark$	$\checkmark$		
This work (Cardinality Constrained Mean-Variance Model)	Mathematical Modeling (Quadratic Programming)	$\checkmark$		$\checkmark$	$\checkmark$	$\checkmark$
This work (MMICC)	Mathematical Modeling (Quadratic Programming)		$\checkmark$	$\checkmark$	$\checkmark$	$\checkmark$

## Literature review

**Figure 2:**
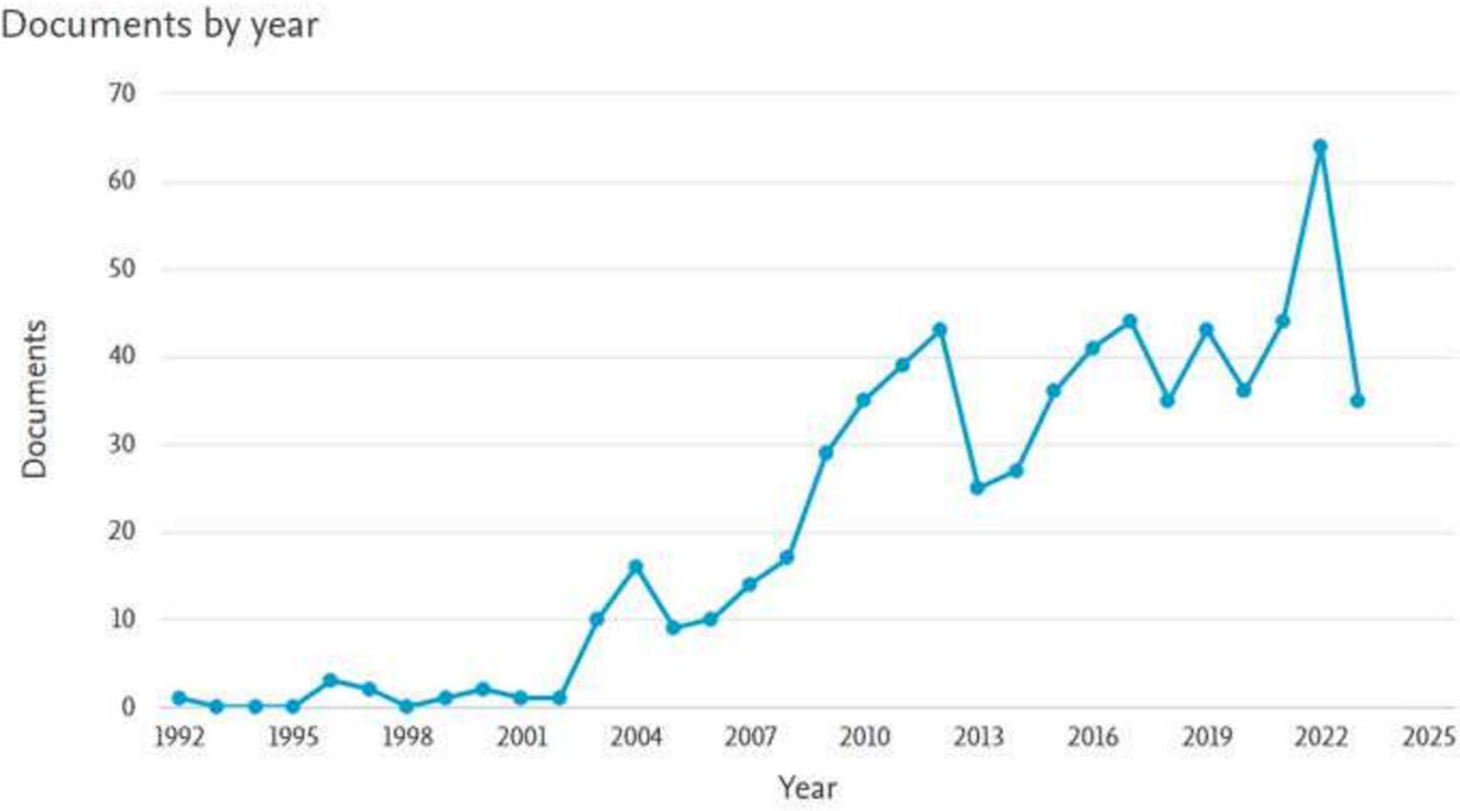
Portfolio optimization publication trend analysis.

The literature review was conducted using “portfolio optimization”, “mean-variance”, and “heuristic” as keywords. Various databases were searched to gather the most relevant information on portfolio optimization, including Elsevier, Springer, and IEEE. The Scopus database, provided by Elsevier, was utilized for the analysis. A range of document types was considered in this research, including articles, conference papers, review articles, book chapters, books, and conference proceedings. The analysis conducted using Scopus is summarized in Fig. 2, which shows the growing interest in portfolio optimization from 1992 to 2023. From Fig. 2, it can be observed that the field of portfolio optimization has been developing as an area of interest over the years. The years from 2009 to 2023 were marked by fluctuations in the field of portfolio optimization. The years 2010 and 2022 were identified as peak periods, indicating that portfolio optimization has been a significant field of research and investigation.

This section presents a detailed review of the different solution methods used in solving portfolio optimization issues, focusing on solving the Unconstrained Portfolio Optimization Problem (UPOP) and Cardinality-Constrained Portfolio Optimization Problem (CCPOP). The methods explored are categorized into three main types: approximate solution methods, hybrid algorithms, and machine learning algorithms, each offering unique approaches to addressing these challenges.

### Approximate solution methods

The approximate solution methods can be divided into two categories, meta-heuristic algorithms and heuristic algorithms.

#### Meta-heuristic algorithms

Meta-heuristic algorithms for solving the UCOP and CCPOP problems are analyzed from two perspectives: Evolutionary Algorithms (EAs) and Swarm Intelligence Algorithms (SIAs).

##### Evolutionary algorithms

Chang et al.^17^ discussed the introduction of the cardinality constraint to the original Markowitz model and its effect on the efficient frontier, which represents the possible tradeoffs between risk and return of the portfolio. Three heuristic algorithms: Genetic Algorithm (GA), Simulated Annealing (SA), and Tabu search (TS) are presented to solve the Unconstrained Portfolio Optimization (UPO) and for the first time the Cardinality Constrained Mean Variance Portfolio Optimization Problem (CCMV-POP).

Woodside-Oriakhi et al.^24^ build upon the work done by Chang et al.^17^ by applying more advanced heuristic algorithms, which include GA, SA, and TA. This resulted in outperforming the solution presented by Chang et al.^17^ within a reasonable timeframe, where the trade-off between computational time and solution quality is common in the algorithmic approaches.

Krink and Paterlini^35^ explored the use of other EA in solving CCMV-POP, which is Differential Evolution for Multi-objective Portfolio Optimization (DEMPO). The paper highlighted the limitations of Quadratic Programming (QP) and Non-Dominated Sorted Genetic Algorithm (NSGA-II) in handling realistic problem properties in portfolio management. DEMPO highlighted its ability to tackle real-world portfolio optimization without oversimplification and achieving satisfactory results in a reasonable time.

Bermudez et al.^36^ presented a novel procedure that extended genetic algorithms beyond traditional optimization to a fuzzy ranking strategy for selecting efficient portfolios of restricted cardinality. This approach modeled the uncertainty of returns using fuzzy quantities and a downside risk function, reflecting investor risk aversion. The study illustrated the performance of this approach with Spanish stock market data, showing the algorithm’s effectiveness in incorporating uncertainty and subjective characteristics into the portfolio selection problem.

Five different Multi-Objective Evolutionary Algorithms (MOEAs) applied to the CCMV-POP were examined by Anagnostopoulos and Mamanis^37^. The author compared the effectiveness of these algorithms, including NSGA-II, SPEA2, NPGA2, PESA, and e-MOEA, against a Single Objective Evolutionary Algorithm (SOEA). The results showed the outperformance of the MOEAs over the SOEA.

##### Swarm intelligence algorithms

Cura^38^ was the first to introduce the Particle Swarm Optimization (PSO) approach in solving portfolio optimization problems. The PSO was developed to solve the CCMV-POP, where the results were compared to those obtained from heuristic methods adopted by Ref.^17^. The study demonstrated the effectiveness of the PSO in portfolio optimization, particularly in terms of success in generating efficient portfolios under lower investment risks.

A Unified Multi-Objective Particle Swarm Optimization (MOPSO) algorithm was discussed by Ref.^39^ for solving the mean-variance portfolio selection problem. The algorithm incorporated an adaptive ranking procedure, which is based on three mechanisms: non-dominated sorting, crowding distance, and a novel one called cost-benefit. The results show that the proposed strategy improved the MOPSO’s performance based on the standard non-dominating sorting implementation. Extensive computational experiments were carried out on five different Portfolio Selection Problems (PSP), each incorporating different constraints. The results showed that this approach achieved highly competitive outcomes across different multi-objective metrics.

Particle Swarm Optimization (PSO) has also been used multiple times in different studies by Refs.^39–42^.

Kao et al.^43^ proposed an approach based on Bacterial Forging Optimization (BFO) by passing through three bacterial forging operations: chemotaxis, reproduction, and elimination dispersal to search for the optimal solution of the portfolio optimization problem under cardinality and bounding constraints. The results were compared to heuristics used by Chang et al.^17^ and Cura^38^ through computational tests on benchmark datasets. The results show that the BFO algorithm outperforms previous heuristics in terms of solution quality and computational time.

Cura^29^ applied the Artificial Bee Colony (ABC) algorithm for solving Markowitz based CCMV model under cardinality and bounding constraints. The paper focused on demonstrating the efficiency and effectiveness of the ABC algorithm compared to other methods such as GA, TS, SA, and others. The study employed data from various stock indices to validate the algorithm’s performance, where the results proved that the ABC outperformed all the heuristics compared with all kinds of investment policies.

Dhaini et al.^28^ presented an adoption of the Squirrel Search Algorithm (SSA) for solving both the unconstrained and the constrained portfolio optimization problem. The study compares the use of SSA along with single metaheuristics, multi-objective metaheuristics, and hybrid metaheuristics. The algorithm demonstrated its superiority in the results of the unconstrained portfolio optimization using extended Mean-Variance and Sharpe models. For the constrained portfolio optimization, the SSA achieved competitive results.

A novel meta-heuristic algorithm based on Mayfly Algorithms (MA) was introduced by Zheng et al.^15^. The algorithm adopted new strategies and features including; cardinality constraint handling strategy, local search strategy, and modifications to crossover operator as the standard MA could not be directly applied for solving single objective CCMV-POP. The algorithm performance was assessed using five performance metrics, and competitive results were demonstrated on datasets of various results.

A time-varying nonlinear programming approach to the mean-variance portfolio selection problem, considering transaction costs and cardinality constraints, was applied using the Beetle Antennae Search (BAS) algorithm by Katsikis et al.^44^. The paper introduced an innovative application of the BAS algorithm to provide online, realistic solutions to this complex financial optimization problem. The effectiveness of this approach was validated through numerical experiments and computer simulations.

A non-deterministic Non-linear Activated Beetle Antennae Search (NABAS) aimed at addressing the complexities of non-convex tax-aware portfolio optimization is introduced by Khan et al.^45^. NABAS addresses the problem faced by traditional methods of getting stuck in suboptimal solutions by maintaining exploration of the search space until a specific gradient estimate threshold is reached, thereby enhancing convergence and avoiding local minima. The algorithm’s performance is compared against other established methods such as BAS, PSO, and GA, demonstrating NABAS’s superior ability to handle non-convex problems.

Quadratic Interpolated Beetle Antennae Search (QIBAS) algorithm, which is an extension of Beetle Antennae Search (BAS) algorithm integrated with Quadratic Interpolation (QI) to enhance its computational efficiency and robustness in higher dimensions, is adopted by Khan et al.^45^ to solve high dimensional portfolio optimization problem with cardinality constraint. The results of QIBAS outperformed other meta-heuristic algorithms such as PSO , BAS, BAS with Zeroing Neural Network (BASZNN), Quantum BAS (QBAS), and GA in terms of convergence speed, expected return, and risk minimization for portfolio selections involving up to 250 companies.

Many other nature-inspired algorithms were used for solving CCMV-POP as Whale Optimization (WO) algorithm^46^, Cat Swarm Optimization (CSO)^47^, Ant Colony Optimization (ACP)^48^, and Bat Algorithm (BA)^49^.

#### Heuristic algorithms

A two-stage approach combining Variable Neighborhood Search (VNS) and Quadratic Programming (QP) was introduced by Akbay et al.^50^. The first stage of the algorithm was responsible for asset selection for the portfolio by adopting VNS, while the second stage determined optimal asset selection through QP. The performance of this approach is compared with other state-of-the-art algorithms using Mean Percentage Error (MEAPE), Median Percentage Error (MEDPE), minimum percentage error (MINPE), and Maximum Percentage Error (MAXPE) which were introduced by Chang et al.^17^ and Mean Euclidean Distance (MEUCD), Mean Return Error (MER), and Variance of Return Error (VRE) performance measures introduced by Cura^38^. The results demonstrated the effectiveness of the approach in solving complex portfolio optimization problems, particularly for portfolios with low risk.

Baykasoglu et al.^51^ developed a Greedy Randomized Adaptive Search Procedure (GRASP) with quadratic programming entitled GRASP-QUAD. The approach was designed in two stages: stock selection based on cardinality constraints, which is based on GRASP, and proportion determination levels of each stock, which is based on a quadratic programming optimization solver, to reduce computational time effectively. The performance of this approach is validated using benchmark datasets, showing the competitiveness and favorable solutions yielded in comparison with some of the best-known results mentioned in the literature as Refs.^11,24,38,52^.

### Hybrid algorithms

Lwin et al.^11^ proposed a hybrid algorithm combining Population-Based Incremental Learning (PBIL) and Differential Evolution (DE) to address portfolio selection problems with cardinality, floor, and ceiling constraints. The algorithm adopted a notable feature of a partially guided mutation and elitist strategy, which enhances the evolution process over the complex and constrained search space. The features adopted were key components contributing to the effectiveness of the Population-Based Incremental Learning and Differential Evolution (PBILDE). The algorithm performance was evaluated using benchmark datasets, and the results indicated superiority in handling compared to traditional methods.

A hybrid algorithm that combines a Multi-objective Evolutionary Algorithm Based on Decomposition (MOEA/D) and Data Envelopment Analysis (DEA) was developed by Zhou et al.^53^. The approach enhanced the initial solution generation created using DEA. The performance of this algorithm, DEA- MOEA/D, is compared with other algorithms such as FDH-MOGA, MOEA/D, and NSGA II across various test functions and portfolio optimization problems. The results indicate that DEA-MOEA/D outperformed the different algorithms not only in test functions but also in portfolio optimization.

A hybridized approach of Artificial Bee Colony (ABC) with Firefly Algorithm (FA) was developed by Ref.^54^ for solving the CCMV portfolio optimization problem. FA is adopted to enhance the algorithm’s overall efficiency due to the slow convergence and the unbalanced tradeoff between exploitation and exploration of the original ABC algorithm. The author showed that this solution approach outperformed state-of-the-art algorithms such as GA, SA, TS, and PSO, especially in the mean Euclidean distance from the standard efficiency frontier.

Khan et al.^55^ proposed a novel framework of the Distributed Beetle Antennae Search (DBAS) algorithm, which combines the swarm-like behavior of Particle Swarm Optimization (PSO) with the updating rules of Beetle Antennae Search (BAS) to address the privacy concerns in multi-portfolio selection problems. The effectiveness and efficiency of DBAS are demonstrated through simulations on real-world stock data from 25 NASDAQ companies over 200 days. The results show that DBAS not only preserved privacy but also outperformed other methods in terms of robustness, computational economy, and time efficiency.

### Machine learning algorithms

Fernandez et al.^52^ applied heuristic algorithms based on Artificial Neural Networks (ANN), specifically using Hopfield networks to solve the CCMV-POP. The paper compared the results obtained with the heuristic algorithms used by Ref.^17^. It was shown that the ANN provided promising results in the instances requiring diversified portfolios with low investment risk.

Xu et al.^56^ proposed a novel algorithm for solving the Cardinality Constrained Portfolio Selection (CCPS) problem. This algorithm, entitled PBIL-CCPS, hybridized Population-Based Incremental Learning (PBIL) with a continuous variant (PBILc) to optimize asset selection and capital allocation. The paper demonstrated the algorithm’s effectiveness by comparing it to GA and PSO, showing PBIL-CCPS’s superior performance in generating portfolios with high expected returns. The research contributes significantly to computational finance, especially in efficiently handling complex portfolio selection problems.

A factor-model-based clustering algorithm is utilized by Jiang et al.^57^ to reduce computational complexity. This approach is distinct in its use of factor models to characterize assets and cluster them into groups, from which representatives are selected to form an efficient portfolio under cardinality constraints. The results demonstrated that incorporating and invoking factor models in finance, clustering analysis, and mixed integer programming models reduced computational burdens and produced meaningful outcomes.

A novel neural network approach is proposed by Cao and Li^58^ to solve portfolio optimization problem under nonconvex cardinalty constraint and transaction cost. The paper proved the global convergence for the proposed model, demonstrating its robustness through numerical experiments. Although the proposed neural network typically identifies one local optimum, it significantly outperforms existing MATLAB routines in terms of solution quality. The experimental results showed a 123.6% reduction in cost compared to average portfolio selections using real stock data from the Dow Jones Industrial Average (DJI) companies in 2006.

**Figure 3:**
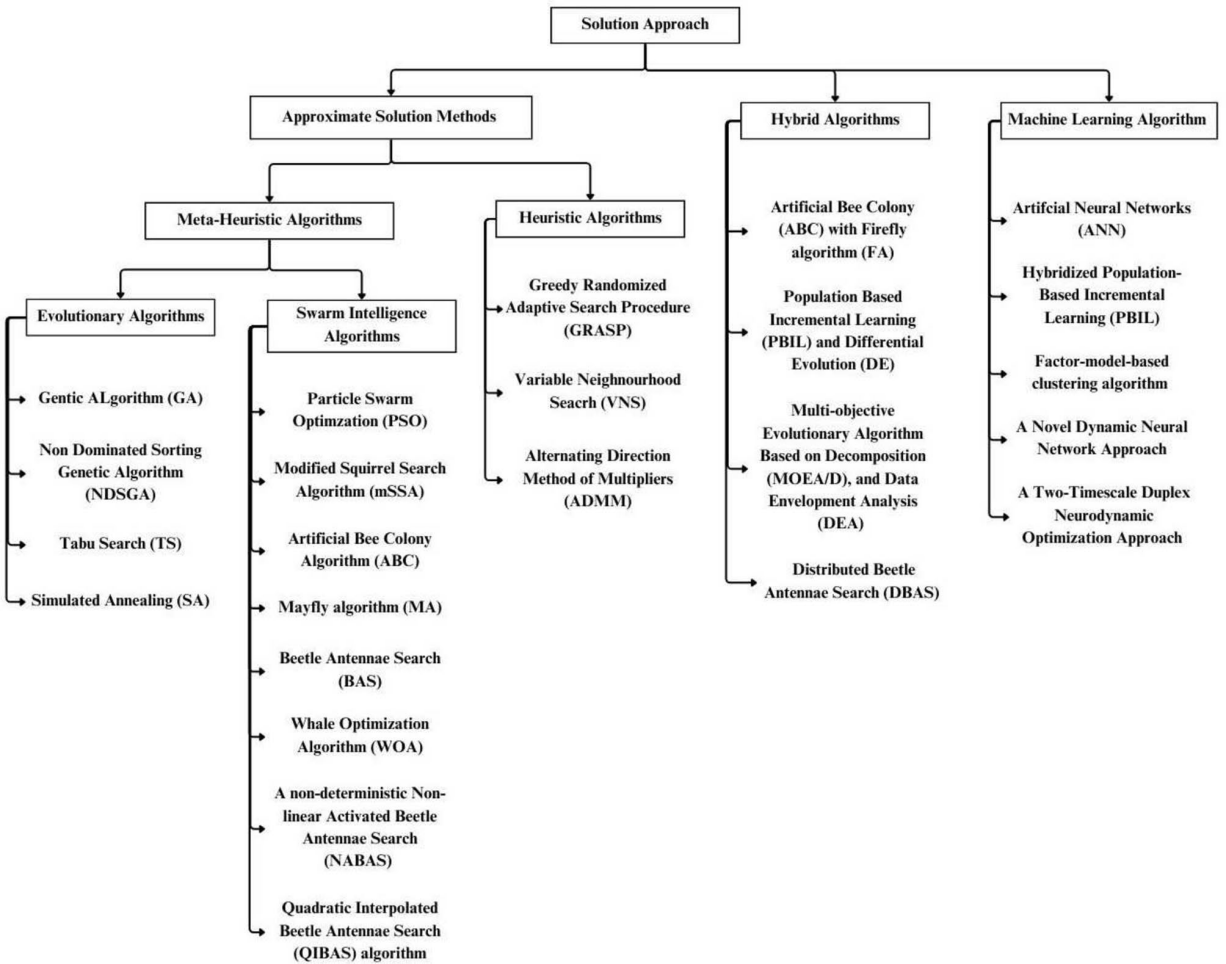
Summarized literature review.

A two-timescale duplex neurodynamic optimization approach to tackle the nonconvex nature of the problem was developed by Leung et al.^59^. The approach reformulated the portfolio selection problem as a biconvex optimization problem, efficiently addressing the complexities and computational demands. Through extensive numerical experiments, the proposed method demonstrates superior performance in achieving optimal portfolio configurations, balancing risk and return, and outperforming traditional methods in terms of computational efficiency and robustness. Figure 3 summarizes the aforementioned approaches.

Based on the review of the literature, the main contributions presented in this paper are:

1. Introducing a novel approach based on BWAPO to solve the Unconstrained Portfolio Optimization Problem (UPOP) and Cardinality Constrained Mean-Variance Portfolio Optimization Problem (CCMV-POP). The performance of the proposed approach has been tested against other approaches from the literature using different data sets with different KPIs.
2. A notable gap in the literature regarding the simultaneous consideration of equality/inequality cardinality, bounding, transaction, and rounding to lots of constraints is identified. To address this, a mathematical model that incorporates all these constraints, offering a comprehensive approach to portfolio optimization is developed.
3. Solving the equality and inequality cardinality Constraint versions of the problem using the BWAPO and the mathematical model, and the quality of the portfolios generated from both versions were compared in each case

## Solution approach

In this section, the two distinct approaches that have been adopted are presented. The first approach involves a sophisticated mathematical model designed to solve the fully constrained mean-variance model. This model comprehensively addresses multiple constraints, including cardinality, bounding, transaction costs, transaction lots, and budget values. The second approach adopts the innovative Black Widow Algorithm for Portfolio Optimization, a method uniquely suited for solving both the unconstrained and cardinality-constrained mean-variance models.

### The proposed mathematical model

The proposed mathematical model is adopted to represent the portfolio optimization problem more practically and realistically. The formulation includes extra constraints such as transaction costs, transaction lots, and a budget limit measured in dollars. Additionally, the cardinality constraint is addressed in two forms within the model: once as an equality constraint and another time as an inequality, further enhancing the model’s alignment with real-world scenarios.

#### Model nomenclature

$$\begin{array}{l l l} i, j &  \in I &  \text {Set of assets}\\ \end{array}$$

- Parameters $$\begin{array}{ll} r_i &  \text {Expected return of asset }i \text {in the portfolio}\\ S_i &  \text {Standard deviation of asset }i\\ \rho _{ij} &  \text {Correlation between asset }i \text {and asset }j \\ K &  Maximum number of assets to be selected in the portfolio\\ L_i &  \text {Lot size specified for asset }i\\ P_i &  \text {Price specified for asset }i\\ c_i &  \text {Transaction cost for asset }i\\ B &  Maximum Budget that cannot be exceeded\\ V &  Minimum allowable value of the portfolio\\ \delta _i &  \text {Maximum allowable weight of asset }i \text {in the portfolio}\\ \varepsilon _i &  \text {Minimum allowable weight of asset} i \text {in the portfolio}\\ \lambda &  \text {Risk avoidance parameter which }\in [0,1]\\ \end{array}$$
- Decision variables
  In this model, the decision variables are categorized into two types: independent and dependent variables, as detailed below:
  1. Independent decision variables
    $$\begin{aligned} ~~~x_i&~~~{\left\{ \begin{array}{ll} 1, &  \text {if}\ \text {asset }i\text { is chosen} \\ 0, &  \text {otherwise} \end{array}\right. }&\end{aligned}$$
    $$\begin{array}{ll} n_i &  \text {Number of assets }i\text { included in the portfolio}\\ t_i &  \text {Number of lots taken from asset }i\\ \end{array}$$
  2. Dependent decision variables $$\begin{array}{ll} R_p &  \text {Expected return of portfolio }P\\ \sigma _p &  \text {Calculated risk of portfolio }P\\ w_i &  \text {Weight of asset }i\\ \end{array}$$
- Objective function
  The objective function (Eq. (11)) uses variance as a measure of risk, aiming to minimize the portfolio’s overall risk. Alongside minimizing the risk, the model also focuses on maximizing the portfolio’s expected return. The expected return is calculated based on historical returns of the assets. The core of the mean-variance model is the trade-off between risk and return. Investors must decide their level of risk tolerance and choose a portfolio that aligns with their investment goals based on variable $\lambda$, where a higher $\lambda$ indicates a greater emphasis on minimizing risk, while a lower $\lambda$ suggests a focus on maximizing returns^19^.
  11 $$\begin{aligned} \min \quad \lambda \left( {\sigma _{p} } \right) - \left( {1 - \lambda } \right) \left( {R_{p} } \right)&\end{aligned}$$
  The portfolio return is represented as shown in Eq. (12)^19^
  12 $$\begin{aligned} R_p&=\sum \limits _{i=1}^I r_iw_ix_i \end{aligned}$$

The portfolio risk is represented as shown in Eq. (13)^19^

13 $$\begin{aligned} \sigma _p&=\sum \limits _{i=1}^I\sum \limits _{j=1}^I w_iw_jCov_{ij} \end{aligned}$$

The Cov$_{ij}$ represents the covariance between each two assets $a_i$ and $a_j$ which is calculated as shown in Eq. (14)

14 $$\begin{aligned} Cov_{ij}&=\rho _{ij}S_iS_j \end{aligned}$$

Therefore, the objective function can be reformulated as in Eq. (15):

15 $$\begin{aligned} \text{ min } ~~ ~~&\lambda \left( \sum \limits _{i=1}^I \sum \limits _{j=1}^I w_iw_jCov_{ij} \right) - (1-\lambda )\sum \limits _{i=1}^I r_iw_ix_i \end{aligned}$$

#### Model formulation

Equation (16) represents the equality cardinality constraint, which restricts the total number of assets in the portfolio. In comparison, Eq. (17) represents the inequality cardinality constraint. In this model, both equality and inequality forms of cardinality constraints are incorporated. The subsequent sections will demonstrate how these two types of cardinality constraints influence the outcomes differently.

16 $$\begin{aligned}{}&\sum \limits _{{i = 1}}^{I} {x_{i} = K}&\end{aligned}$$

17 $$\begin{aligned}{}&\sum \limits _{i=1}^I x_i\le K&\end{aligned}$$

Equation (18) is the boundary constraint that imposes a lower and upper limit on the weights of the assets.

18 $$\begin{aligned}{}&\varepsilon _i x_i \le w_i \le \delta _i x_i, ~ \forall i \in I&\end{aligned}$$

Equations (19) and (20) represent the rounding to lots constraint, which enforces that the decision variable $n_i$ (representing asset number) is an integer value. The formula effectively rounds the quantities of assets to integer values.

19 $$\begin{aligned}{}&n_i \ge \frac{w_iB}{P_ix_i}, ~ \forall i \in I&\end{aligned}$$

20 $$\begin{aligned}{}&L_i = \frac{n_i}{t_ix_i}, ~ \forall i \in I&\end{aligned}$$

Equation (21) is a conditional constraint that adapts the “big- M” method which effectively activates or deactivates the $t_i$ value based on the value of the binary variable $x_i$.

21 $$\begin{aligned}{}&t_i \le Mx_i&\end{aligned}$$

Equation (22) guarantees that the total investment including the transaction cost of asset *i* and price of the asset *i* in the portfolio does not exceed a certain budget (B).

22 $$\begin{aligned}{}&\sum \limits _{i=1}^I n_ix_i(c_i+P_i)\le B&\end{aligned}$$

Equation (23) guarantees that the total investment including the transaction cost of asset *i* and price of the asset *i* in the portfolio value does not fall under a certain value.

23 $$\begin{aligned}{}&\sum \limits _{i=1}^I n_ix_i(c_i+P_i)\ge V&\end{aligned}$$

The model has been verified and validated using a data set of 501 assets as will be explained in the “Results” section.

### Black Widow Algorithm for Portfolio Optimization

**Figure 4:**
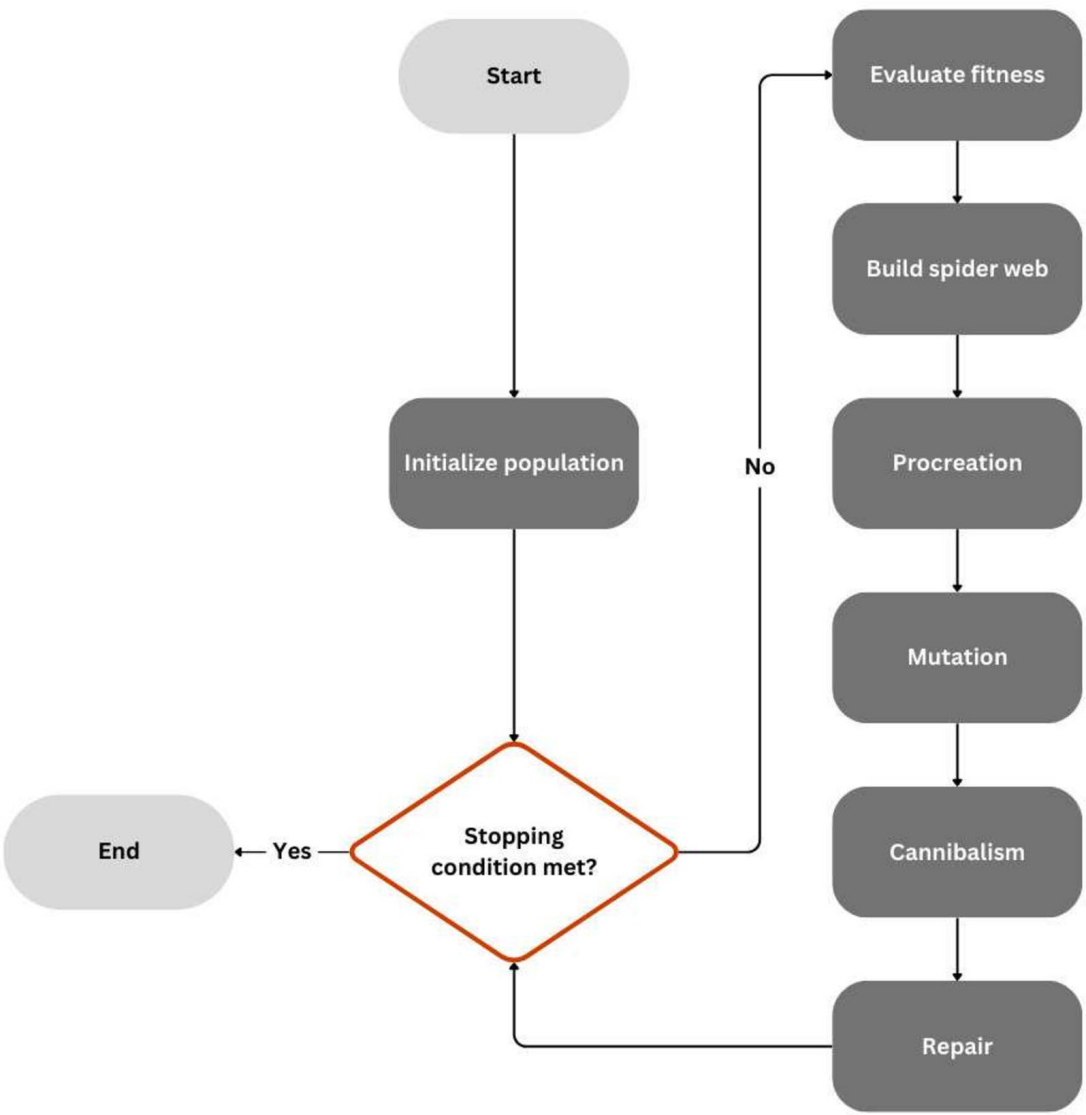
The proposed BWAPO.

**Figure 5:**
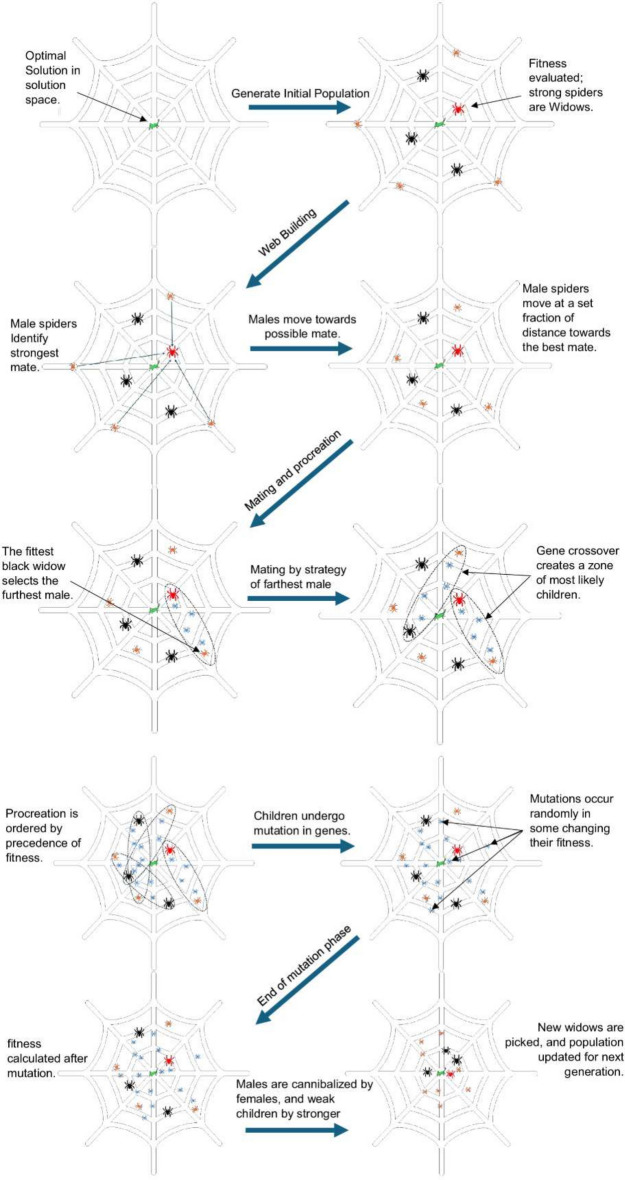
Visual representation of the proposed BWAPO.

The Black Widow Algorithm for Portfolio Optimization (BWAPO) is a relatively new population-based meta-heuristic algorithm inspired by the evolution process of spider population^16^. The algorithm mirrors the behavioral patterns of black widow spiders by destroying weak spiders (solutions) and guiding the population of artificial agents to the optimal solution following an artificial spider agent. Modifications have been made to the original algorithm to allow for solution space exploration. Figure 4 portrays the proposed algorithm flowchart, Fig. 5 shows a visual representation of the proposed algorithm, while Algorithm 1 represents the pseudo-code for the proposed algorithm.

**Figure:**
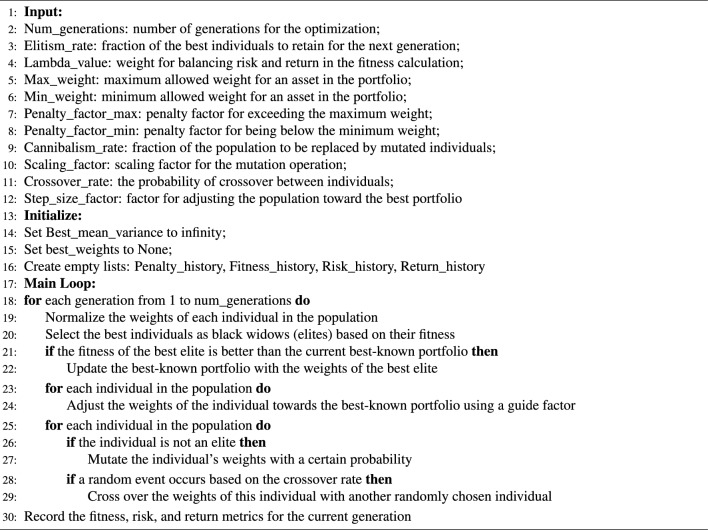
**Algorithm 1**. Pseudo code of Black Widow Algorithm for Portfolio Optimization.

#### Exploration and exploitation in the Black Widow Algorithm

The BWO, inspired by black widow spiders, balances exploration (broad search) and exploitation (refinement within promising areas):

1. Exploration Mechanisms:
  Mating and Cannibalism: Generates diverse candidate solutions (portfolios) and eliminates weaker ones. This maintains high-quality solutions and avoids redundancy.
  Mutation: Introduces random changes, preventing premature convergence and ensuring wide coverage of potential solutions.
2. Exploitation Mechanisms:
  Selective Survival: Retains the fittest solutions, refining the population based on fitness (better risk-adjusted returns in financial markets).
  Intensification Around Elites: Focuses search around top-performing portfolios, optimizing promising areas.

#### Relevance to financial market complexity

1. High Dimensionality and Large Datasets: Financial markets involve numerous assets, leading to high-dimensional problems:
  Broad Search Capability: The BWO’s diverse solution generation and mutations ensure the solution space is extensively explored, crucial for finding optimal portfolios.
2. Multi-objective optimization and constraint handling: Portfolio optimization involves balancing multiple objectives and includes constraints like weight limits:
  Trade-off Management: The BWO navigates trade-offs between maximizing returns and minimizing risk through exploration and targeted refinement.
  Effective Constraint Management: The BWO generates diverse solutions, ensuring feasible ones are found, and refines them within constraints.

#### Initialization

The population is constituted by a matrix *W* of weights of size $[N \times n]$ where *n* is the population size of spiders or portfolios, while *N* is the number of assets in the dataset.

$$\begin{aligned} W=  { \begin{bmatrix}w_{11} &  w_{12} &  \cdots &  w_{1n}\\ w_{21} &  \cdots &  \cdots &  \cdots \\ \cdots &  \cdots &  \cdots &  \cdots \\ w_{N1} &  \cdots &  \cdots &  w_{Nn}\end{bmatrix} } \\\end{aligned}$$

encapsulating the weights assigned to each asset in the portfolios. Each weight ($w_{Nn}$) is drawn from the real numbers set *R*.

#### Evaluating fitness

The objective function of each portfolio is defined by its mean-variance characteristics, aiming to strike a balance between returns and risks. In mathematical terms, for a given portfolio represented as a weight vector *w*, the mean-variance objective function can be expressed as follows:

24 $$\begin{aligned} f(w)&=\lambda \cdot w^T \beta w - (1-\lambda ) \cdot w^{T}R \end{aligned}$$

where $w^T$ denotes the transpose of the weight vector, $\beta$ signifies the covariance matrix, and *R* represents the vector of asset returns. The parameter $\lambda$ controls the trade-off between risk and return. After the objective function is calculated, the population is sorted, and elites are picked out as the top performers within the spiders according to their objective function.

#### Building web

After the fitness of portfolios is evaluated, the elites are selected according to their objective function score, the population is sorted, and the best-performing spider, according to its objective function, is picked. This spider is considered the fittest widow $P^{Best}$, attracting possible mates. A web is constructed with male spiders moving towards $P^{Best}$ at a fixed fraction of the distance between the possible mates and $P^{Best}$^60^. Consider the following matrix *W* of spiders:

$$\begin{aligned} W=  { \begin{bmatrix}w_{11} &  w_{12} &  \cdots &  w_{1n}\\ w_{21} &  \cdots &  \cdots &  \cdots \\ \cdots &  \cdots &  \cdots &  \cdots \\ w_{N1} &  \cdots &  \cdots &  w_{Nn}\end{bmatrix} } \\\end{aligned}$$

To calculate the distance from each spider to $P^{Best}$ of weights:

$$ P^{{Best}} = \left[ {\begin{array}{*{20}l} {w_{1}^{{Best}} } \\ {w_{2}^{{Best}} } \\ \cdots \\ {w_{N}^{{Best}} } \\ \end{array} } \right] $$

The difference between $P^{Best}$ and each possible mate in the population is calculated. So, it can be calculated using matrix subtraction by repeating $P^{Best}$ *N* number of times in a matrix. The result is a matrix of all of the distances between each possible mate and $P^{Best}$ as follows:

$${ \begin{bmatrix}w_1^{Best} &  w_1^{Best} &  \cdots &  w_1^{Best}\\ w_2^{Best} &  \cdots &  \cdots &  \cdots \\ \cdots &  \cdots &  \cdots &  \cdots \\ w_N^{Best} &  \cdots &  \cdots &  w_N^{Best}\end{bmatrix} } -  { \begin{bmatrix}w_{11} &  w_{12} &  \cdots &  w_{1n}\\ w_{21} &  \cdots &  \cdots &  \cdots \\ \cdots &  \cdots &  \cdots &  \cdots \\ w_{N1} &  \cdots &  \cdots &  w_{Nn}\end{bmatrix} }=  { \begin{bmatrix}Dist_{11} &  Dist_{12} &  \cdots &  Dist_{1n}\\ Dist_{21} &  \cdots &  \cdots &  \cdots \\ \cdots &  \cdots &  \cdots &  \cdots \\ Dist_{N1} &  \cdots &  \cdots &  Dist_{Nn}\end{bmatrix} } \\$$

This resultant distance matrix can then be multiplied by a fraction of the distance, $\varepsilon$, which is a constant parameter then the resultant matrix is then added to the matrix of spiders to adjust their weights towards the best spider:

$$ \varepsilon  = \left[ {\begin{array}{*{20}l}    {Dist_{{11}} } \hfill & {Dist_{{12}} } \hfill &  \cdots  \hfill & {Dist_{{1n}} } \hfill  \\    {Dist_{{21}} } \hfill &  \cdots  \hfill &  \cdots  \hfill &  \cdots  \hfill  \\     \cdots  \hfill &  \cdots  \hfill &  \cdots  \hfill &  \cdots  \hfill  \\    {Dist_{{N1}} } \hfill &  \cdots  \hfill &  \cdots  \hfill & {Dist_{{Nn}} } \hfill  \\   \end{array} } \right] + \left[ {\begin{array}{*{20}l}    {w_{{11}} } \hfill & {w_{{12}} } \hfill &  \cdots  \hfill & {w_{{1n}} } \hfill  \\    {w_{{21}} } \hfill &  \cdots  \hfill &  \cdots  \hfill &  \cdots  \hfill  \\     \cdots  \hfill &  \cdots  \hfill &  \cdots  \hfill &  \cdots  \hfill  \\    {w_{{N1}} } \hfill &  \cdots  \hfill &  \cdots  \hfill & {w_{{Nn}} } \hfill  \\   \end{array} } \right] = \left[ {\begin{array}{*{20}l}    {w_{{11}}^{{New}} } \hfill & {w_{{12}}^{{New}} } \hfill &  \cdots  \hfill & {w_{{1n}}^{{New}} } \hfill  \\    {w_{{21}}^{{New}} } \hfill &  \cdots  \hfill &  \cdots  \hfill &  \cdots  \hfill  \\     \cdots  \hfill &  \cdots  \hfill &  \cdots  \hfill &  \cdots  \hfill  \\    {w_{{N1}}^{{New}} } \hfill &  \cdots  \hfill &  \cdots  \hfill & {w_{{Nn}}^{{New}} } \hfill  \\   \end{array} } \right] $$

where $w_{Nn}$ represents the updated population after the movement towards $P^{Best}$.

#### Procreation

To apply the crossover between parents, a matrix *C* of random numbers is generated having the size $\frac{N \times n}{2}$. The numbers generated belong to the range [0,1]. The black widows well as the mates are split into two matrices of size $\frac{N \times n}{2}$. *Parent*1 represents the black widows, which are extracted from matrix *W* representing the first half of the sorted matrix. The opposite happens to *parent*2 which represents black widow mates, which are weaker in nature and are extracted from the second half of the matrix *W*.

$$ C = \left[ {\begin{array}{*{20}l}    {c_{{11}} } \hfill & {c_{{12}} } \hfill &  \cdots  \hfill & {c_{{1n}} } \hfill  \\    {c_{{21}} } \hfill &  \cdots  \hfill &  \cdots  \hfill &  \cdots  \hfill  \\     \cdots  \hfill &  \cdots  \hfill &  \cdots  \hfill &  \cdots  \hfill  \\    {c_{{N1}} } \hfill &  \cdots  \hfill &  \cdots  \hfill & {c_{{Nn}} } \hfill  \\   \end{array} } \right],Parent1 = \left[ {\begin{array}{*{20}l}    {w_{{11}} } \hfill & {w_{{12}} } \hfill &  \cdots  \hfill & {w_{{1(n/2)}} } \hfill  \\    {w_{{21}} } \hfill &  \cdots  \hfill &  \cdots  \hfill &  \cdots  \hfill  \\     \cdots  \hfill &  \cdots  \hfill &  \cdots  \hfill &  \cdots  \hfill  \\    {w_{{N1}} } \hfill &  \cdots  \hfill &  \cdots  \hfill & {w_{{N(n/2)}} } \hfill  \\   \end{array} } \right],Parent2 = \left[ {\begin{array}{*{20}l}    {w_{{1((n/2) + 1)}} } \hfill & {w_{{1((n/2) + 2)}} } \hfill &  \cdots  \hfill & {w_{{1n}} } \hfill  \\    {w_{{2((n/2) + 1)}} } \hfill &  \cdots  \hfill &  \cdots  \hfill &  \cdots  \hfill  \\     \cdots  \hfill &  \cdots  \hfill &  \cdots  \hfill &  \cdots  \hfill  \\    {w_{{N((n/2) + 1)}} } \hfill &  \cdots  \hfill &  \cdots  \hfill & {w_{{Nn}} } \hfill  \\   \end{array} } \right] $$

The offspring matrix *O* is generated by applying the crossover mask to the parent matrices:

25 $$\begin{aligned} O&= C \cdot Parent1 + (1-C) \cdot Parent2&\end{aligned}$$

$$\begin{aligned} O =  { \begin{bmatrix}O_{11} &  O_{12} &  \cdots &  O_{1(n/2)}\\ O_{21} &  \cdots &  \cdots &  \cdots \\ \cdots &  \cdots &  \cdots &  \cdots \\ O_{N1} &  \cdots &  \cdots &  O_{N(n/2)} \end{bmatrix} } \\\end{aligned}$$

This matrix *O* represents the next generation of spiders, incorporating diverse genetic traits from both parents based on the percentage present in matrix *C*. Where matrix *C* is regenerated once for each child.

- Mating attraction
  1. Financial Context Adaptation: In the Black Widow Algorithm (BWO), the mating attraction process drives portfolios to move towards the best-performing ones. This mechanism ensures that portfolios inherit desirable traits from the top portfolios, facilitating the exploration of the search space.
  2. Exploration and Convergence: By encouraging different portfolios to gravitate towards the best portfolio, the algorithm explores various combinations of assets. This not only helps in discovering diverse and potentially optimal asset mixes but also aids in convergence by iteratively refining the population towards higher-performing solutions.

#### Mutation

The algorithm utilizes a differential evolution mutation operation, first used in Ref.^61^. The mutation is governed by the mutation rate (MR), which involves randomly selecting spiders for mutation. The mutation operation is expressed as follows:

26 $$\begin{aligned} P^{New}&= P^{Best}_{t} + S \times (P^{r1}_{t}-P^{r2}_{t})&\end{aligned}$$

where $P^{Best}_{t}$ is the best portfolio in generation *t*, $P^{r1}_{t}$ and $P^{r2}_{t}$ are randomly selected portfolios from the current population, $(r1\ne r2)$, *T* is the total number of generations, and $S=\frac{t}{T}$ is the mutation operator that increases with the number of iterations.

- Differential evolution mutation strategy
  1. Financial Context Adaptation: The differential evolution (DE) mutation strategy introduces variations by combining strong portfolios with random ones. This adaptive mutation enhances diversity and exploration within the population.
  2. Optimality and Diversity: By creating mixtures of random portfolios with strong portfolios, the algorithm adds a level of exploration necessary for escaping local optima. This strategy ensures that the algorithm continues to explore new areas of the solution space while maintaining the potential for finding optimal solutions.

#### Cannibalism

Cannibalism occurs as a mechanism to introduce diversity and evolution within the spider population. This process involves selecting a subset of spiders to undergo genetic mutation, simulating the notion of self-cannibalism to drive adaptation. The operations associated with cannibalism can be expressed in matrix form, emphasizing the element-wise calculations involved. After the procreation occurs, the fitness of individuals is reevaluated, and then the spider population is sorted according to fitness, with the weakest spiders being set for cannibalization by stronger spiders. This is identified and denoted according to a cannibalism operator (*CR*). The larger the cannibalism operator, the fewer survivors will remain based on their fitness. Where *CR* of 0.6 would mean 60% of the population would be destroyed.

#### Repair mechanism and feasibility

##### Penalty functions

To guide the optimization, penalty functions are introduced to enforce constraints on portfolio weights. These penalties penalize portfolios for exceeding maximum weights, being below minimum weights, and deviating from the cardinality constraint. The penalties are added to the objective function to influence the optimization process.

Cardinality penalty: To ensure that the cardinality penalty is not broken during the algorithm’s processing, we apply a penalty to portfolios that exceed the cardinality as follows:

27 $$\begin{aligned} x_{i}&={\left\{ \begin{array}{ll} 1, &  \text {if}\ {w_{i}\ge 0} \\ 0, &  \text {otherwise} \end{array}\right. }&\end{aligned}$$

where $x_i$ is a binary variable representing whether asset *i* is included in the portfolio or not.

28 $$\begin{aligned} Cardinality~penalty&= \left| \sum _{i=1}^{N}(x_i)-K\right| \times Penalty&\end{aligned}$$

where *K* is the cardinality limit set by the portfolio. This equation calculates the cardinality penalty which is applied to a portfolio’s objective function when the number of assets exceeds or is below the cardinality limit. Applying this method means that the cardinality penalty that breaks the cardinality limit with more assets is more penalized.

Exceeding maximum weight penalty: Let *W* be the matrix representing portfolios, $w_{i}$ representing the weight of asset *i* in a portfolio. The penalty terms are calculated as:

29 $$\begin{aligned} w_{i}-w_{max}&={\left\{ \begin{array}{ll} w_{i}-w_{max}, &  \text {if}\ {w_{i}-w_{max}\ge 0} \\ 0, &  \text {otherwise} \end{array}\right. }&\end{aligned}$$

This equation represents a penalty term for exceeding the maximum weight $w_{max}$ for a particular asset *i*. If the weight exceeds the maximum allowed, the penalty is the difference between the actual weight and the maximum weight; otherwise, the penalty is zero. The penalty term is then aggregated over all assets *N* and $P_{max}$ representing the penalty for each portfolio.

30 $$\begin{aligned} P_{max}&= \sum _{i=1}^{N}\left( (w_{i}-w_{max})\times Penalty\right)&\end{aligned}$$

Below minimum weight penalty: Similarly, this equation represents a penalty term for being below the minimum weight $w_{min}$ for a particular asset i is below the minimum allowed, the penalty is the difference between the minimum weight and the actual weight; otherwise, the penalty is zero.

31 $$\begin{aligned} w_{min}-w_{i}&={\left\{ \begin{array}{ll} w_{min}-w_{i}, &  \text {if}\ {w_{min}-w_{i}\ge 0} \\ 0, &  \text {otherwise} \end{array}\right. }&\end{aligned}$$

The penalty term is then aggregated over all assets *N* and $P_{min}$ representing the penalty for each portfolio.

32 $$\begin{aligned} P_{min}&= \sum _{i=1}^{N}\left( (w_{min}-w_{i})\times Penalty\right)&\end{aligned}$$

These penalty equations are essential in portfolio optimization to enforce constraints on asset weights, ensuring they do not exceed maximum or fall below minimum values. The penalty factor *Penalty* quantifies the cost associated with violating these constraints. The objective is to minimize the total penalty, thus encouraging the algorithm to find solutions that adhere to the specified weight boundaries. The penalties are then summed up and added to the objective function.

##### Ensuring feasibility within constraints

Throughout the optimization process, elites (top-performing portfolios) are chosen based on their fitness, considering both the objective function and penalty terms. The various operations, such as web-building, crossover, and mutation, are carried out to explore the solution space while ensuring adherence to the specified constraints. The repair operation can be mathematically expressed as follows:

Let *I* be the matrix containing the indices of the assets of highest weights in each portfolio. The repaired portfolio $P_{repaired}$ is obtained by setting all elements outside these indices to zero:

33 $$\begin{aligned} w_{i}&={\left\{ \begin{array}{ll} w_{i}, &  \text {if}\ {w_{i}\in I} \\ 0, &  \text {otherwise} \end{array}\right. }&\end{aligned}$$

##### Cardinality and budget constraints with penalty function

1. Penalty Function Integration: The addition of a penalty function to the mean-variance objective ensures that portfolios violating constraints are naturally considered weaker. The penalty function imposes a cost on portfolios that break cardinality and budget constraints.
2. Exploration within Constraints: This approach allows the population to explore areas of strength within the constraints, as portfolios that adhere to constraints are favored. If portfolios fail to comply during the mating attraction phase, they incur penalties and are less likely to survive.
3. Natural Selection: Portfolios that consistently break constraints are deemed weak and die off, ensuring that constraint-violating portfolios do not remain within the gene pool. This natural selection process guarantees that the population evolves towards feasible and optimized solutions.

##### Combined constraint handling

The BWO’s combination of exploration and exploitation mechanisms, along with the penalty function for constraint management, ensures effective portfolio optimization:

1. Constraint-Aware Initialization: The population is initialized with portfolios that meet cardinality and budget constraints, providing a feasible starting point.
2. Penalty Function Application: Throughout the algorithm’s iterations, the penalty function continually assesses and penalizes constraint violations, guiding the population towards compliance.
3. Selective Survival: The selective survival process ensures that only portfolios that adhere to all constraints and demonstrate high fitness survive, driving the population towards optimized solutions.

#### Overall complexity

The components and their respective complexities are broken down, then analyze how these components contribute to the overall complexity. The overall complexity of the algorithm is dominated by the most computationally intensive steps, which involve matrix operations and sorting. Specifically:

- Mean-Variance Calculation: $O(N\times n^2)$
- Parent Selection: Includes sorting, resulting in $O(N \times n^2 + NlogN)$

Among these, the quadratic term $O(N\times n^2)$ is the dominant term, as $n^2$ scales faster than *NlogN*. Thus, the overall time complexity of the portfolio optimization algorithm is: $O(N\times n^2)$, Where:

- *N* is the population size.
- *n* is the number of assets in the dataset.

This indicates that the algorithm’s complexity scales quadratically with the number of assets and linearly with the population size.

## Computational experiments

### Experimental procedures

To assess the effectiveness of the Black Widow Algorithm for Portfolio Optimization, the Unconstrained Efficient Frontier (UEF) and the Cardinality Constrained Efficient Frontier (CCEF) are plotted. The results have been computed by setting the parameters to $K=10$, $\varepsilon _i=0.01$ ($i=1, 2, \dots , N$) and $\delta _i=1$ ($i=1, 2, \dots , N$) and with varied risk aversion parameters $\lambda$, incrementing by 0.02. This means conducting 51 runs to capture the efficient frontier, recording the optimal solution in each instance. All the runs were computed on a workstation with a processor: Intel(R) Xeon(R) Gold 6230R CPU @ 2.10GHz (2 processors) with 128 GB RAM.

In our proposed approach, the population size and the number of generations are determined based on the number of assets, *N*, consistent with the methodology used in previous studies for comparison. Specifically, we set the population size to $20\sqrt{N}$ and the number of generations to $50\sqrt{N}$^28^.

The mathematical model parameters were set as follows: The budget (*B*) was set at $10,000, indicating the total amount of money available for investment. Transaction costs were calculated as 1% of the asset price ($$c_i = 0.01p_i$$), which will be deducted from the budget for each transaction made. The minimum and maximum weights for all the assets in the portfolio were set at 0.01 and 1, respectively. The lot size parameter is 1, suggesting that assets can be bought in individual units. The portfolio must maintain a minimum value (*V*) of $7000, ensuring that the portfolio’s worth does not fall below this threshold. Each run of the optimization process for each $$\lambda $$ value was set for a time limit of 300 s, ensuring efficient computation and decision-making. Lastly, the cardinality limit was set to be $$K=0.2N$$ (number of assets in the portfolio). These parameters collectively guide the investment strategy, balancing risk, diversification, cost, and time efficiency.

### Benchmark data sets

This paper used five datasets from the OR-Library, a source extensively cited in prior CCMV-POP studies, as originally presented in Ref.^62^. These datasets, comprising Hong Kong Hang Seng, German DAX 100, British FTSE 100, US S &P 100, and Japanese Nikkei 225, offer weekly price data from March 1992 to September 1997. These datasets and their UEF are publicly accessible at http://people.brunel.ac.uk/~mastjjb/jeb/orlib/portinfo.html. These five instances are the most frequently utilized in solving portfolio optimization problems using the CCMV model through a metaheuristic approach. This comprehensive data set was instrumental in evaluating the efficacy of our proposed algorithm.

For the mathematical model, Yahoo finance daily close prices are used, which can be publicly accessed from https://shorturl.at/sHQX6. The dataset includes detailed daily trading information from January 2010 to December 2016. It consists of opening, closing, high, and low prices, along with trading volumes for various 501 stocks, each represented by its symbol. The calculation of the return on an asset from day *t* to day $t+1$ is given by the formula^63^:

34 $$\begin{aligned}{}&r_t = \frac{Price_{t+1}-Price_t}{Price_t}&\end{aligned}$$

where, $P_{t+1}$ and $P_t$ represent the closing prices of the asset on day $t+1$ and day *t*, respectively. Then the expected return for asset *i* is calculated using the following formula^63^:

35 $$\begin{aligned}{}&E(r_i) = \frac{\Sigma _{t=1}^mr_t^i}{m}&\end{aligned}$$

where $r_t^i$ denotes the return of asset *i* across different time intervals, and m represents the total number of these periods. The datasets considered for the experimental runs are listed in Table 2.

**Table 2:** Benchmark datasets.

Model	Index	No. of assets	Country
Cardinality constrained MV model	Hang Seng	35	Hong Kong
	Dex 100	83	Germany
	FTSE 100	85	United Kingdom
	S &P 100	98	USA
	Nikkei 225	225	Japan
The proposed mathematical model	S &P 500	501	USA

### Performance indicators

Several performance metrics are adopted to evaluate and compare the performance of the proposed approach with various algorithms used in portfolio optimization. These metrics fall under the convergence-based indicators category, which assesses how closely the algorithms’ outcomes align with the ideal Pareto front and their overall diversity.

#### Mean return error and variance of return error

The MRE and VRE presented by Ref.^52^ measure the vertical and horizontal deviations, respectively, of the obtained solutions from specific levels of risk or return, indicating the closeness of the approximation to the theoretical Pareto optimal front.

#### Percentage deviations

The Mean Percentage Error (MPE) and Median Percentage Error (MedPE) are calculated following the approach proposed by Ref.^17^. These metrics evaluate the average and median percentage deviations from the optimal solution, offering insights into the precision of the algorithm’s efficient frontier.

#### Generational Distance (GD)

As mentioned by Ref.^64^, GD quantifies the average Euclidean distance between the obtained frontier and the actual Pareto front. A smaller GD value indicates a closer match to the optimal efficient frontier, signifying a more effective algorithm. In this paper, the introduced black widow approach is evaluated against prior studies, as detailed in Table 3, sourced from Ref.^28^.

**Table 3:** Prior studies selected for comparative analysis.

Approach/algorithm	Portfolio Optimization Variant	Model	Metric	Work	Year
GA, TS, SA	UPO, CCPO	Extended MV model	MPE, MedPE	^17^	2000
PSO	CCPO	Extended MV model	MRE, VRE	^38^	2009
GA, PSO, PBIL	UPO	MV model	MPE, MedPE	^56^	2010
GA, TS, SA	CCPO	Extended MV model	MRE, VRE	^24^	2011
PSO-HNN	CCPO	Extended MV model	MRE, VRE	^65^	2012
DE, PBIL, PBILDE	UPO, CCPO	Extended MV model	MPE,MedPE	^11^	2013
FA, uFA	UPO, CCPO	Extended MV model	MPE,MedPE	^66^	2014
NSGA-II, PESA-II, SPEA2, MOPSO	CCPO	Extended MV model	GD, MRE, VRE	^18^	2014
NSGA-II, PESA-II, SPEA2, MOPSO, NS-MOPSO	CCPO	Extended MV model	GD, MRE, VRE	^67^	2014
ABC-FS	CCPO	Extended MV model	GD, MRE, VRE	^54^	2014
ICPSO, HIS	CCPO	Extended MV model	MRE, VRE	^68^	2014
GRASP-QUAD	CCPO	Extended MV model	MRE, VRE	^51^	2015
ABC	CCPO	Extended MV model	MPE, MedPE, GD, MRE, VRE	^26^	2017
GI-ABC	CCPO	Extended MV model	GD, MRE, VRE	^69^	2018
ARMOPSP	UPO, CCPO	Extended MV model	MPE, MedPE, GD MRE, VRE	^39^	2019
CACO	CCPO	Extended MV model	MPE, MedPE, GD MRE, VRE	^2^	2020
VNS+QP	CCPO	Extended MV model	MPE, MedPE, GD MRE, VRE	^50^	2020
mSSA	CCPO	Extended MV model	MPE, MedPE, GD MRE, VRE	^28^	2021

### Experimental results

In this section, we evaluate the performance of the unconstrained Black Widow Algorithm for Portfolio Optimization (BWAPO) by comparing its results with those from the unconstrained mathematical model. This comparison aims to validate the effectiveness of the BWAPO. Additionally, the performance of the constrained BWAPO is assessed against established algorithms. The section then explores the impact of incorporating the inequality cardinality constraint into the BWAPO, comparing it with the version of BWAPO that employs the equality cardinality constraint. This comparison aims to determine which constraint delivers a higher return and a more effective solution. Finally, the proposed constrained mathematical model is introduced, along with an examination of both the equality and inequality cardinality constraints.

#### Unconstrained portfolio optimization results

**Figure 6:**
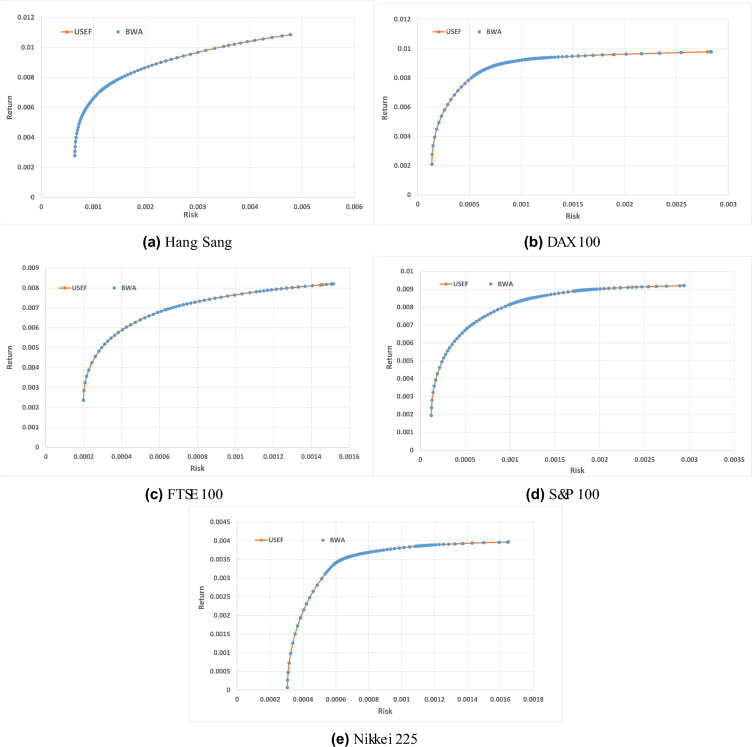
Comparing the results of the BWAPO against the USEF for different datasets.

To assess the efficiency of the Black Widow Algorithm for Portfolio Optimization (BWAPO) in solving the portfolio optimization problem. The heuristic efficient frontier obtained from the BWAPO is plotted against the Unconstrained Standard Efficient Frontier (USEF). The USEF represents the optimal portfolios obtained without any restrictions imposed on the cardinality (*K*), and the lower and upper bounds of the weights are set to be 0 and 1, respectively. As shown in Fig. 6, the heuristic frontiers are very close to the standard frontiers tested on the five benchmark instances, Hang Seng, DAX 100, FTSE 100, S &P 100 and Nikkei from OR library.

The figures show that the suggested heuristic algorithm closely approaches the Unconstrained Standard Efficient Frontier (USEF) across the five instances. Consequently, the BWAPO yields near-optimal portfolio solutions, closely aligning with the optimal outcomes. This indicates that BWAPO demonstrates remarkable convergence towards the UEF.

Table 4 compares the BWAPO proposed approach with other single meta-heuristic algorithms according to the MPE and MedPE performance metrics. The proposed approach outperforms the other algorithms in FTSE100, while it is ranked second in Hang Seng, DAX100, and S &P 100.

Table 5 compares the computational results of different hybrid meta-heuristic algorithms, which are also used by Ref.^28^ for comparison. The BWAPO outperforms the other algorithms in FTSE100, S &P100, and Nikkei 225 except for the small instance datasets Hang Seng, and Dax 100.

**Table 4:** MPE and MedPE comparison against unconstrained single meta-heuristics.

Instances	Number of assets	Metric	GA	TS	SA	GA	PSO	DE	FA	uFA	mSSA	BWAPO
Hang Seng	35	MPE	0.0202	0.8973	0.1129	0.0191	0.1422	0.0280	0.2302	0.0193	0.0000487	0.000261809
		MedPE	0.0165	1.0718	0.0161	0.0166	$<0.00001$	0.0000028	2.7835	0.0162	0.000004	0.0000140448
Dax 100	85	MPE	0.0136	3.5645	0.0394	0.0350	1.1044	0.0089	2.7835	0.0115	0.0023	0.003828028
		MedPE	0.0123	2.7816	0.0033	0.0124	$<0.00001$	0.000021	2.1035	0.0103	0.00003	0.001361428
FTSE 100	89	MPE	0.0063	3.2731	0.2012	0.0109	1.143	0.0049	2.9056	0.0089	0.0048	**0.0046117807**
		MedPE	0.0029	3.0238	0.0426	0.0020	0.0084	0.0000019	2.6791	0.0010	0.000077	0.00217448511
S &P 100	98	MPE	0.0084	4.4281	0.2158	0.0430	2.0249	0.0137	–	–	0.0044	0.0048866782
		MedPE	0.0085	4.2781	0.0142	0.0085	0.5133	0.000003	–	–	0.000036	0.0021737671
Nikkei 225	225	MPE	0.0085	15.9163	1.7681	0.3715	8.1781	0.0606	–	–	0.00680	0.0323145717
		MedPE	0.0084	14.2668	0.8107	0.0068	4.7023	0.000026	–	–	0.000435	0.0211272416
Avg.		MPE	0.0114	5.6158	0.4675	0.0959	2.5185	0.0603	–	–	0.00367	0.007275383

Significant values are in bold.

**Table 5:** MPE and MedPE comparison against unconstrained hybrid and multi-objective meta-heuristics.

Instances	Number of assets	Metric	PBIL	PBIL	PBIL-DE	ARMOPSO	mSSA	BWAPO
Hang Seng	35	MPE	0.0003	0.2385	0.0002	0.0002	**0.0000487**	0.000261809
		MedPE	$<0.0001$	0.0257	$<0.0001$	$<0.0001$	**0.000004**	0.0000140448
Dax 100	85	MPE	0.0023	1.1849	0.0052	0.0050	**0.0023**	0.003828028
		MedPE	$<0.0001$	0.4292	$<0.0001$	$<0.0001$	0.00003	0.001361428
FTSE 100	89	MPE	0.0186	0.9813	0.0059	0.0064	0.0048	**0.0046117807**
		MedPE	$<0.0001$	0.0799	$<0.0001$	$<0.0001$	0.000077	0.00217448511
S &P 100	98	MPE	0.0137	1.2361	0.0078	0.0073	**0.0044**	0.0048866782
		MedPE	< **0.0001**	0.1443	$<0.0001$	0.0038	0.000036	0.0021737671
Nikkei 225	225	MPE	0.0606	3.7411	0.2733	0.0721	**0.00680**	0.0323145717
		MedPE	$<0.0001$	2.0514	$<0.0001$	0.0244	0.000435	0.0211272416
Avg.		MPE	0.0191	1.4763	0.0584	0.0182	**0.00367**	0.007275383

Significant values are in bold.

#### Constrained portfolio optimization results

This section showcases the outcomes of the problem’s cardinality-constrained version, with the following parameters: $K=10$, $\varepsilon = 0.01$, and $\gamma = 1$. The performance of the BWAPO in CCMV-POP is evaluated against different hybrid and multi-objective meta-heuristic approaches as shown in Tables 6, 7 and 8.

**Table 6:** Comparison of computational outcomes for MPE and MedPE for equality constrained POP.

Instances	Metric	GA	PBIL	DE	PBIL	PBILDE	MCS	ABC	CACO	ARMO PSO	VNS$+$ QP	mSSA	BWAPO
Hang Seng	MPE	1.0974	1.1026	1.215	1.3894	1.1431	1.0462	1.0953	1.0965	1.0520	1.0964	**0.0155**	0.0198736712
	MedPE	1.2181	1.2190	1.2331	1.578	1.2390	1.1946	1.2181	1.2181	0.7917	1.2155	0.01512	**0.0093162766**
Dax 100	MPE	2.5424	2.5163	3.3077	2.5129	2.4251	2.4402	2.3258	2.313	2.1570	2.3125	**0.01459**	0.0916451996
	MedPE	2.5466	2.5739	2.741	2.5850	2.5866	2.4501	2.5678	2.5587	2.0184	2.5630	**0.01048**	0.0718856909
FTSE 100	MPE	1.1076	0.996	1.3651	1.319	0.9706	1.4550	0.8481	0.8451	0.9128	0.8453	**0.00955**	0.0849528958
	MedPE	1.0841	1.0841	1.0975	1.1204	1.084	1.0781	1.0841	1.0841	0.6642	1.0840	**0.00548**	0.031404318
S &P 100	MPE	1.9328	2.232	3.2208	2.4722	1.6386	1.6974	1.2930	1.2930	1.6176	1.2649	**0.01849**	0.197139938
	MedPE	1.2244	1.1536	1.5970	1.2096	1.1692	1.2244	1.1369	1.1323	1.2170	1.1323	**0.01887**	0.052839344
Nikkei 225	MPE	0.7961	1.0017	1.8934	0.7554	0.5972	0.6635	0.5781	0.5781	0.6178	0.5904	**0.01651**	0.158225006
	MedPE	0.6133	0.5854	1.6428	0.6592	0.5896	0.6635	0.5856	0.5854	0.2273	0.5857	**0.01642**	0.0823542730
Avg.	MPE	1.49526	1.56972	2.2004	1.68978	1.35492	1.46046	1.22806	1.22514	1.27144	1.2219	**0.014928**	0.110367

Significant values are in bold.

**Table 7:** Comparison of computational outcomes for VRE and MRE for equality constrained POP.

Instances	Metric	PSO-HNN	ICPSO	IHS	PESAII	SPEA2	NSGAII	MOPSO	GRASP	ARMO PSO	mSSA	BWAPO
Hang Seng	VRE	2.590	1.900	1.804	1.523	1.487	1.326	1.284	1.640	1.151	4.67	**0.486316952**
	MRE	0.733	0.641	0.648	0.762	0.689	0.647	0.602	0.606	0.574	0.427	**0.228112409**
Dax 100	VRE	5.758	7.206	7.380	9.282	8.243	7.121	6.754	6.759	6.293	13.38	**1.249024090**
	MRE	1.147	1.188	1.043	2.221	1.592	1.263	1.267	1.277	1.098	1.118	** 0.4318424096**
FTSE 100	VRE	5.414	3.381	3.248	5.238	3.765	2.987	2.812	2.430	2.184	5.2	**0.634406938**
	MRE	0.309	0.324	0.320	0.402	0.365	0.333	0.325	0.324	0.307	**0.206**	0.363012920
S &P 100	VRE	5.416	4.589	3.902	7.012	5.432	3.763	3.476	2.521	2.406	10.76	**1.859792004**
	MRE	**0.292**	0.896	0.948	2.423	1.211	0.732	0.702	0.906	0.771	1.35	1.034247444
Nikkei 225	VRE	4.778	1.841	1.602	3.098	2.042	1.123	0.987	**0.836**	0.901	9.23	2.240880885
	MRE	0.704	0.433	0.403	1.231	0.865	0.432	0.327	0.418	**0.322**	1.86	0.6245352363

Significant values are in bold.

**Table 8:** Comparison of computational outcomes for GD, VRE and MRE for equality constrained POP.

Instances	Metric	GA	PBIL	DE	PBIL	PBILDE	MCS	ABC	CACO	ARMO PSO	VNS$+$ QP	mSSA	BWAPO
Hang Seng	GD	0.004	0.004	0.004	0.0049	0.0004	0.0003	0.0002	0.0001	0.0001	0.0001	0.0000778	**0.00004099**
	VRE	1.6441	1.6578	1.6628	2.2421	1.3952	1.2387	1.2295	1.6395	1.6397	1.6397	4.6738	**0.45505466**
	MRE	0.6072	0.6107	0.6238	0.7427	0.5289	0.4715	0.4703	0.6085	0.6058	0.6058	0.42762	**0.22140521**
Dax 100	GD	0.0076	0.0082	0.0078	0.009	0.0009	0.0009	0.0009	0.0001	0.0001	0.0001	0.0001	**4.21999E−05**
	VRE	7.218	9.0309	8.5485	6.8588	7.2649	7.2569	7.1981	6.7806	6.7583	6.7583	13.38	**0.842620781**
	MRE	1.2791	1.9078	1.2817	1.5885	1.35229	1.3786	1.2885	1.278	1.2767	1.2767	1.118	**0.376393169**
FTSE 100	GD	0.002	0.0021	0.0021	0.0022	0.0003	0.0004	0.0003	0	0	0	0	0.000058998
	VRE	2.866	4.0123	3.8205	3.0596	2.6721	2.7085	2.6354	2.435	2.4349	2.4349	5.2059	**0.54931695**
	MRE	0.3277	0.3298	0.3304	0.364	0.31872	0.3121	0.3109	0.3186	0.3252	0.3252	**0.206**	0.339647604
S &P 100	GD	0.0041	0.0041	0.0041	0.0052	0.0001	0.0003	0.0001	0.0001	0.0001	0.0001	0.0001	0.0001
	VRE	3.4802	5.7139	5.4247	3.9136	3.7598	3.6026	3.5991	2.5255	2.5105	2.5105	10.76	**1.115312761**
	MRE	1.2258	0.7125	0.8416	1.404	0.95292	0.8993	0.881	**0.7044**	0.9072	0.9072	1.3552	1.004281983
Nikkei 225	GD	0.0093	0.001	0.001	0.0019	0	0	0	0	0	0	0.0001	0.0000179758
	VRE	1.2056	1.2431	1.2017	2.4274	1.69823	1.2015	1.2011	0.8191	0.8561	0.8561	9.23	**0.699217656**
	MRE	5.3266	0.4207	0.4126	0.7997	0.67192	0.4892	0.4713	0.4233	0.4217	0.4217	1.86	**0.408474492**

Significant values are in bold.

In Table 6, the BWAPO outperforms all the algorithms for the MedPE in the Hang Seng dataset and comes in second in performance after the mSSA for all the performance metrics for all the datasets. Tables 7 and 8 present the results using metrics MRE and VRE for comparison. In Table 7, the results of metric MRE for the BWAPO outperform the rest of the eleven approaches presented in Hang Sang and Dex 100, got the fourth-best result for Nikkei 225. The BWAPO is outperformed in larger data sets by other FTSE 100 and S &P 100 approaches by mSSA and PSO-HNN, respectively. For Metric VRE, BWAPO outperforms all the other algorithms in all the benchmark datasets. The low values of VRE indicate that the resulting frontier points of the BWAPO are very close to the optimal.

Table 8 presents the comparison with other algorithms. Considering MRE, our approach yields the most favorable outcomes in comparison to other methods for three out of five data sets, namely Hang Seng, DAX 100, and Nikkei 225. Regarding VRE, the proposed algorithm demonstrated superior performance, surpassing all other algorithms in the comparison across all data sets. GD is employed alongside MRE and VRE as a measure of performance. Analyzing computational outcomes based on GD values reveals that the frontier achieved by our algorithm is nearest to the optimal, recording the lowest GD values for Hang Seng and DAX 100. It ties with ABC-FS, Ufa, GI-ABC, ABC, CACO, VNS + QP, and mSSA for the S &P 100. However, it ranks second to other algorithms for the Nikkei data set.

From the results the BWAPO shows strong performance in specific scenarios, particularly in small datasets such as Hang Seng and DAX 100. It excels in minimizing variance-related errors (VRE) across all benchmark datasets, indicating its robustness in maintaining a low variability of results. The algorithm performs exceptionally well in certain datasets (Hang Seng, DAX 100, and Nikkei 225) regarding Mean Relative Error (MRE), but its effectiveness varies across different datasets. It demonstrates a somewhat weaker performance in larger datasets such as the FTSE 100 and S &P 100, where other algorithms such as mSSA and PSO-HNN outperform it.

In terms of Generational Distance (GD), BWAPO achieves the closest to optimal solutions in Hang Seng and DAX 100, indicating its effectiveness. However, its performance is less optimal in the Nikkei dataset, where it ranks second to other algorithms. While BWAPO often outperforms many other algorithms, it is not universally superior. The algorithm seems less effective in larger datasets, such as the larger FTSE 100 and S &P 100 datasets.

#### Cardinality constrained BWAPO results

**Figure 7:**
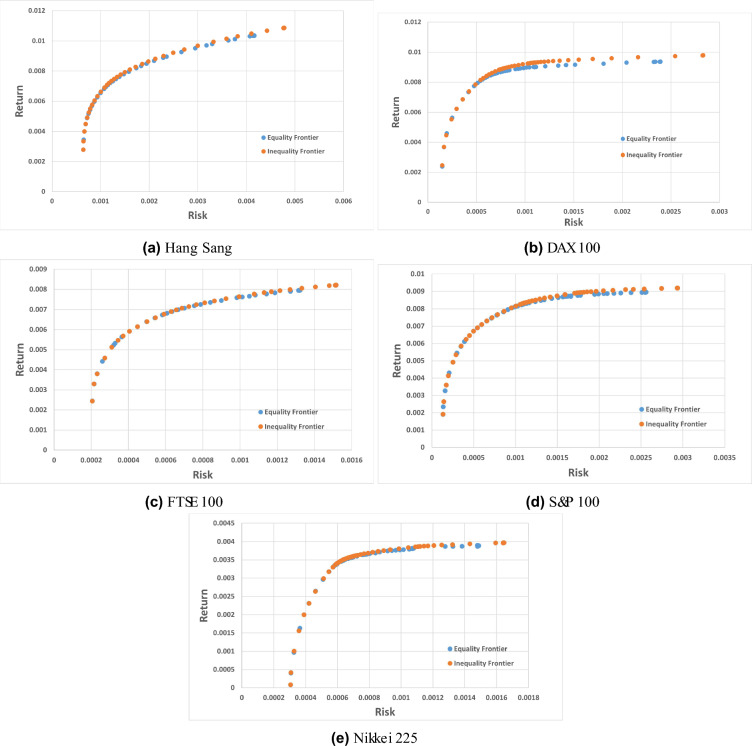
Comparing inequality and equality cardinality constraint BWAPO for different datasets.

The BWAPO utilizes the inequality cardinality constraint to address the CCMV-POP problem, and its results are evaluated against those achieved using the equality cardinality constraint. It is important to note that in the BWAPO with the inequality cardinality constraint, the minimum weight limit is set at 0, rather than 0.01, allowing for the assignment of zero weights. Figure 7 represents the efficient frontiers of both the inequality and equality cardinality constraint.

Figure 7 shows that the inequality frontier consistently sits slightly above the equality frontier, indicating a higher return for a given level of risk. This suggests that even with a relatively small number of assets, the inequality BWAPO offers a greater reward for the risk encountered, though the difference is not clearly noticeable.

In Fig. 7c,d there is a high level of overlap between the equality and inequality frontiers especially at low-risk levels. However, the inequality frontier offers marginally higher returns when compared to the equality frontier at higher risk levels.

The most significant gap between the equality and inequality frontiers appears in Fig. 7e, with the inequality frontier offering higher returns across the entire risk spectrum. The dispersion of points in the inequality frontier suggests a wider range of potential outcomes, reflecting greater variability and potential for higher returns at the expense of higher risk.

These observations across the different benchmark datasets show the advantage of inequality constraint as the number of assets increases. While smaller portfolios perform similarly under equality and inequality constraints, larger portfolios perform significantly better when inequality constraints are applied. This suggests that for investors willing to accept higher levels of risk, inequality constraints may provide a more profitable return profile, particularly as the assets become more diversified.

The proposed mathematical model results

In this section, the outcomes derived from the mathematical model mentioned in section are presented. The results are obtained by applying both equality and inequality cardinality constraints to determine which method yields better results. The model was executed on 18 data files, beginning with ten assets, incrementing by ten assets up to 100 assets, followed by increments of 50 assets from 100 to 500. The selection of assets in each data file was made randomly to ensure a diverse and unbiased sample. The displayed graphs represent portfolios comprising 60, 150, 300, and 450 assets.

**Figure 8:**
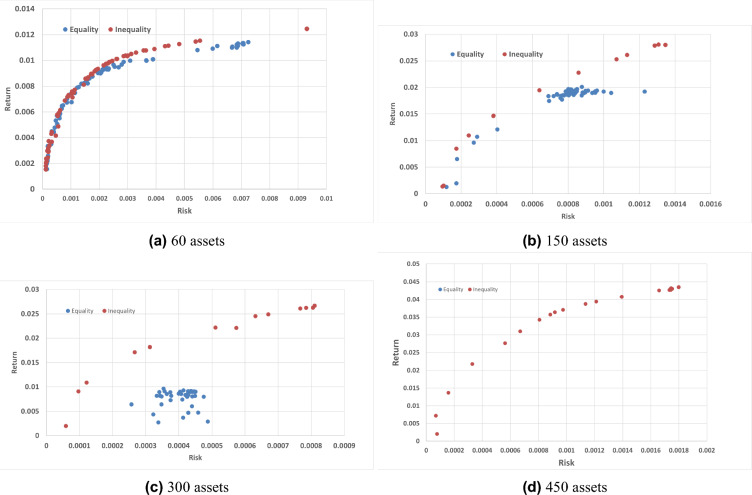
The proposed mathematical model comparison on different sizes of assets.

In Fig. 8a, The efficient frontier for both equality and inequality cardinality constraints begins to converge at higher levels of risk. However the inequality constraint model offers slightly higher returns for the same level of risk compared to the equality constraint model, particularly at lower levels of risk. In Fig. 8b the difference in returns between the two methods appears more pronounced, with the inequality constraint method generally offering higher returns for a given level of risk. Also, the spread of portfolio options is wider in the inequality method, suggesting more diversity in the risk-return profiles available.

The portfolios for the equality constraint in Fig. 8c are more centrally located, suggesting a narrower range of risk-return portfolios. On the other hand, the inequality constraint has a more spread-out portfolio, indicating a wider range of potential outcomes This indicates that the equality constraint model is more restrictive, leading to portfolios that have similar levels of risk and return.

For the 450 and 500 assets datasets, the equality constraint model was unable to find a feasible solution under the given constraints as shown in Fig. 8d of 450 assets. This is due to the constraints being too restrictive or the model reaching a limitation in handling a large number of assets. On the other hand, the inequality constraint model’s ability to offer solutions even at this scale demonstrates its flexibility and the possibility of achieving higher returns, although with potentially increased risk.

The results indicate that the inequality cardinality constraint method provides a broader set of feasible solutions across all portfolio sizes. Specifically for the large assets portfolios, where the equality constraint method failed to yield any results, the inequality method provided potential portfolios, which might suggest better adaptability or flexibility of the inequality method in handling larger, more complex portfolios.

## Conclusion and future work

The BWAPO’s efficiency is benchmarked against the Unconstrained Standard Efficient Frontier (USEF), and it closely approaches the USEF, indicating near-optimal portfolio solutions. The BWAPO shows strength in datasets such as the FTSE 100 and competes well against other meta-heuristic algorithms.

The constrained Black Widow Algorithm for Portfolio Optimization (BWAPO) demonstrates strong performance, particularly in small datasets such as the Hang Seng and DAX 100. The BWAPO is effective at minimizing variance-related errors (VRE) across all benchmark datasets, showing robustness and consistency. However, its effectiveness varies with different datasets, showing some limitations in larger datasets such as the FTSE 100 and S &P 100. The ability of the BWAPO to closely match the optimal frontier points, as indicated by the low Generational Distance (GD) values, confirms its effectiveness in portfolio optimization under cardinality constraints. These results suggest that while BWAPO is a capable algorithm for portfolio optimization, its effectiveness can vary depending on the dataset size.

For the inequality cardinality Black Widow Algorithm for Portfolio Optimizations, it reveals that with a smaller number of assets, the inequality constraints slightly edge out the equality constraints, offering higher returns for the same level of risk. As the portfolio size grows, this advantage becomes more evident, particularly at higher risk levels, with the performance gap most evident Nikkei 225 dataset. This suggests a strategic benefit to using inequality constraints for larger, more diverse portfolios, especially for investors who are comfortable with higher risk.

For the mathematical model that tests both equality and inequality cardinality constraints across 18 data files, with asset counts ranging from 10 to 500. It is concluded that inequality constraints generally offer a wider range of feasible solutions and potential for higher returns, especially in larger asset portfolios where equality constraints might be too restrictive, or the model may not perform optimally. Particularly for portfolios with 450 and 500 assets, only the inequality method could provide feasible solutions, highlighting its flexibility and adaptability for managing larger, more complex portfolios.

Future improvements may be focused on enhancing the algorithm’s performance for solving the cardinality-constrained portfolio optimization problem, particularly for large datasets. Additionally, the incorporation of the constraints in the BWAPO, which are encountered in the mathematical model, may also be considered in future work.

The BWAPO’s efficiency is benchmarked against the Unconstrained Standard Efficient Frontier (USEF), and it closely approaches the USEF, indicating near-optimal portfolio solutions. The BWAPO shows strength in datasets such as the FTSE 100 and competes well against other meta-heuristic algorithms.

## Data Availability

All data used during this study are included in this published article.
